# *N6*-methyladenosine-induced ERRγ triggers chemoresistance of cancer cells through upregulation of ABCB1 and metabolic reprogramming

**DOI:** 10.7150/thno.40144

**Published:** 2020-02-10

**Authors:** Zhuojia Chen, Long Wu, Jiawang Zhou, Xinyao Lin, Yanxi Peng, Lichen Ge, Cheng-Ming Chiang, Hui Huang, Hongsheng Wang, Weiling He

**Affiliations:** 1 Guangdong Key Laboratory of Chiral Molecule and Drug Discovery, School of Pharmaceutical Sciences, Sun Yat-sen University, Guangzhou, Guangdong 510006, China; 2 Sun Yat-sen University Cancer Center; State Key Laboratory of Oncology in South China; Collaborative Innovation Center for Cancer Medicine, Guangzhou 510060, China; 3 Institute of Human Virology, University of Maryland School of Medicine, Baltimore, MD 21201, USA; 4 Department of Clinical Laboratory, Jinling Hospital, Nanjing University School of Medicine, 305 East Zhongshan Road, Nanjing 210002, China; 5 Simmons Comprehensive Cancer Center, Department of Pharmacology, and Department of Biochemistry, University of Texas Southwestern Medical Center, 5323 Harry Hines Boulevard, Dallas, Texas 75390, USA; 6 Cardiovascular Department, The Eighth Affiliated Hospital, Sun Yat-sen University, Shennan Middle Road 3025#, Shenzhen, 518033, China; 7 Department of Gastrointestinal Surgery, The First Affiliated Hospital, Sun Yat-sen University, Guangzhou, Guangdong, 510080, China

**Keywords:** Chemoresistance, ERRγ, *ABCB1*, * CPT1B*, FAO

## Abstract

**Background**: Drug resistance severely reduces treatment efficiency of chemotherapy and leads to poor prognosis. However, regulatory factors of chemoresistant cancer cells are largely unknown.

**Methods**: The expression of estrogen receptor related receptors (ERRs) in chemoresistant cancer cells are checked. The roles of ERRγ in chemoresistance are confirmed by* in vitro* and *in vivo* studies. The mechanisms responsible for ERRγ-regulated expression of *ABCB1* and *CPT1B* are investigated.

**Results**: The expression of ERRγ is upregulated in chemoresistant cancer cells. Targeted inhibition of ERRγ restores the chemosensitivity. ERRγ can directly bind to the promoter of *ABCB1* to increase its transcription. An elevated interaction between ERRγ and p65 in chemoresistant cells further strengthens transcription of *ABCB1*. Further, ERRγ can increase the fatty acid oxidation (FAO) in chemoresistant cells via regulation of *CPT1B,* the rate-limiting enzyme of FAO. The upregulated ERRγ in chemoresistant cancer cells might be due to increased levels of N6-methyladenosine (m^6^A) can trigger the splicing of precursor *ESRRG* mRNA.

**Conclusions**: m^6^A induced ERRγ confers chemoresistance of cancer cells through upregulation of *ABCB1* and *CPT1B.*

## Introduction

Chemotherapy using one or more anticancer drugs is the major strategy for cancer treatment, particularly for patients with advanced and/or metastatic tumors that cannot undergo surgery 1. It is generally preferred in developing countries due to high costs of targeted therapies 2. However, many patients gradually develop resistance to progressive chemotherapy, resulting in treatment failure that has become a serious clinical problem in cancer therapy. One important feature of chemoresistance is that cancer cells often become resistant to not just one drug, but also to different drugs. This is known as multidrug resistance (MDR) and will seriously affect the treatment efficiency.

Several mechanisms involved in chemoresistance have been identified in the last few decades 3-5. ATP-binding cassette (ABC) transporters with a family of 48 human members can regulate the absorption, disposition and elimination of drugs to mediate chemoresistance 6. ATP binding cassette subfamily B member 1 (*ABCB1*), which encodes multidrug resistance protein-1 (MDR-1)/P-glycoprotein (P-gp), is one of the best studied molecules in drug resistance 3. It can confer cancer cell resistance to numerous anticancer agents such as doxorubicin (Dox), taxol (Tax), colchicine, vincristine and even tyrosine kinase inhibitors 7.

Recently, emerging evidence indicates that metabolic properties of chemoresistant cancer cells diverge significantly from those of their parental cells 8-10. Dysregulation of glucose metabolism, fatty acid synthesis and glutaminolysis have been linked to therapeutic resistance in cancer treatment 11. Fatty acids (FAs) are important cellular energy resources utilized through FA oxidation (FAO), which has been shown to be involved in cancer stem cell self-renewal and chemoresistance of breast 10 and gastric 12 cancers. Inhibition of FAO can repress stemness and *in vivo* growth of cancer cells 13, 14. However, the roles and regulatory factors of FAO in chemoresistant cancer cells are largely unknown.

Estrogen receptor related receptors (ERRs), which include ERRα/β/γ, are orphan nuclear receptors and share sequence homology with estrogen receptor α (ERα) 15. It has been reported that ERRα is involved in chemotherapy resistance of osteosarcoma cells 16. ERRγ is a crucial mediator of multiple endocrine and metabolic signals 17 and mediates tamoxifen (TAM) resistance of invasive lobular breast cancer, in which knockdown of ERRγ restores TAM sensitivity 18. Androgen-dependent repression of ERRγ reprograms metabolic properties of prostate cancer 19, whereas miR-378 can inhibit the expression of ERRγ to suppress tricarboxylic acid cycle (TCA) gene expression and oxygen consumption as well as an increase in lactate production 20. All these data suggest that ERRs are likely involved in chemoresistance of cancer cells.

We found that ERRγ was significantly upregulated in chemoresistant cancer cells, with knockdown of ERRγ restoring the chemosensitivity. Mechanistically, ERRγ can mediate the chemoresistance of cancer cells via upregulation of *ABCB1* and facilitation of FAO. Our results identify a new macromolecule that may serve as a predictive marker of chemotherapy and as an effective target for overcoming chemoresistance.

## Results

### ERRγ is upregulated in chemoresistant cancer cells

**Figure 1:**
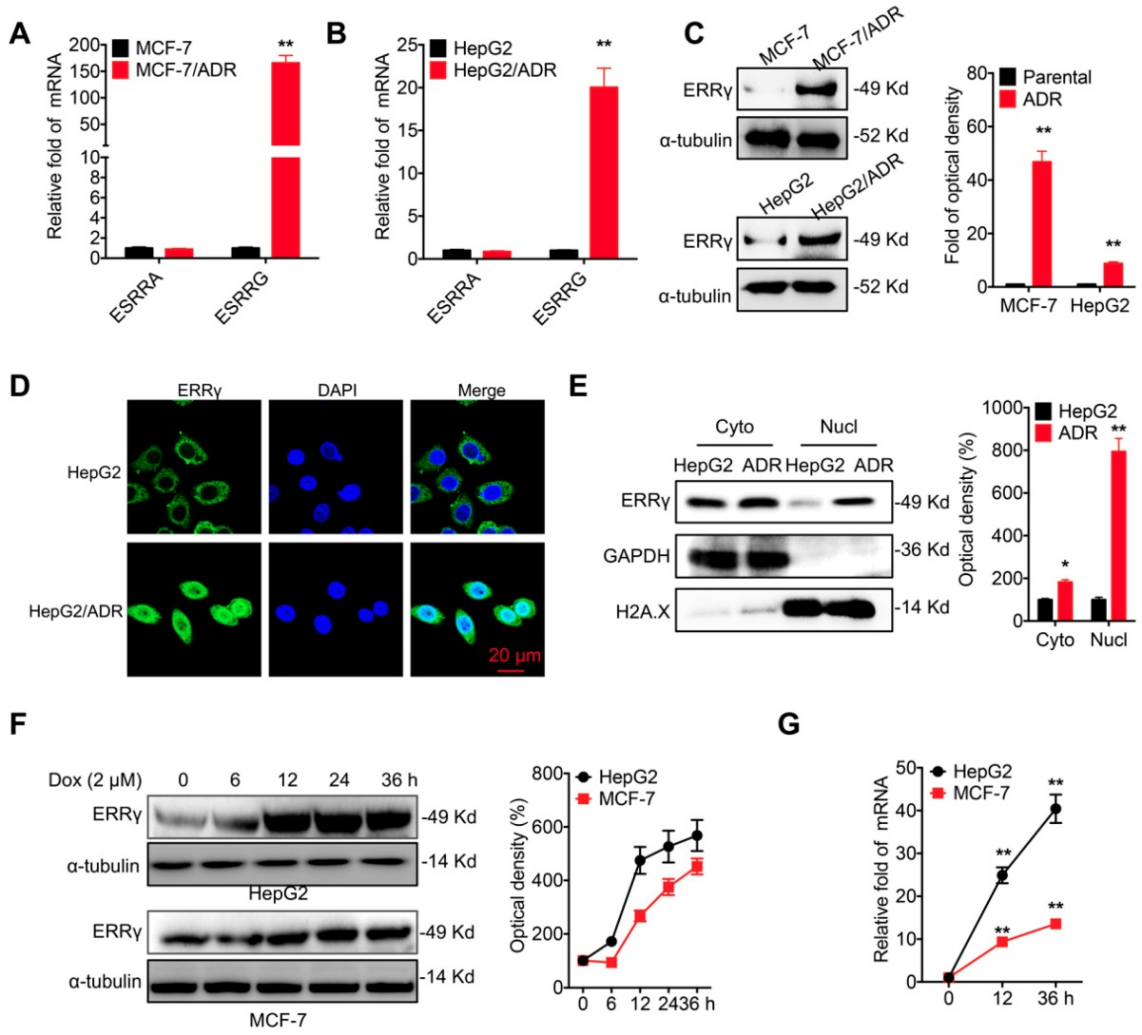
** ERRγ is upregulated in chemoresistant cancer cells.** (A&B) Expression of ERRα (*ESRRA*) and ERRγ (*ESRRG*) measured in MCF-7/ADR (A), HepG2/ADR (B), and their corresponding parental cells by qRT-PCR; (C) Protein levels of ERRγ in MCF-7/ADR, HepG2/ADR, and their corresponding parental cells measured by Western blot analysis (left) and quantitatively analyzed (right); (D) Subcellular expression of ERRγ in HepG2/ADR and HepG2 cells visualized by confocal imaging; (E) The subcellular expression of ERRγ in HepG2/ADR and HepG2 cells was checked by Western blot analysis (left) and quantitatively analyzed (right); (F) Cells were treated with Dox (2 μM) for the indicated times, then the protein expression of ERRγ was checked by Western blot analysis (left) and quantitatively analyzed (right); (G) Cells were treated with Dox (2 μM) for the indicated times, then the mRNA expression of ERRγ was checked by qRT-PCR. Data were presented as means ± SD from three independent experiments. *p<0.05, **p< 0.01 compared with control.

The chemoresistance of breast (MCF-7/ADR) and liver (HepG2/ADR) cancer cells was confirmed by evaluation of Dox sensitivity and compared with that of their corresponding parental cells (Figure S1A and S1B). A potential role of ERR signals in chemoresistance was then assessed by quantifying the mRNA levels of ERRα (*ESRRA*) and ERRγ (*ESRRG*). Quantitative (q) RT-PCR showed enhanced mRNA levels of ERRγ in MCF-7/ADR cells as compared to that in MCF-7 cells (Figure 1A). Increased expression of ERRγ was also observed in HepG2/ADR cells as compared to that in HepG2 cells (Figure 1B). An elevated protein level of ERRγ was also observed in MCF-7/ADR and HepG2/ADR cells by Western blot analysis (Figure 1C). In contrast, the protein levels of ERRα had no clear difference between chemoresistant and parental cells (Figure S1C). An increased mRNA level of ERRγ was also observed in other chemoresistant lines, including MCF-7/Tax (resistance to Tax), MDA-MB-231/Tax, and A549/Tax cells as compared to their corresponding parental cells (Figure S1D). Western blot analysis further confirmed that the protein level of ERRγ was increased in MCF-7/Tax and MDA-MB-231/Tax as compared to their controls (Figure S1E). Confocal microscopy showed that the expression of ERRγ was mainly located within the cytoplasm of HepG2 cells, while the expression and nuclear accumulation of ERRγ were increased in HepG2/ADR cells (Figure 1D). An increased nuclear localization of ERRγ in HepG2/ADR cells was confirmed by subcellular fractionation and Western blot analysis (Figure 1E) and further validated in MCF-7/ADR cells (Figure S1F). The expression levels of ERRα/γ in cancer cells were also examined following Dox treatment. Western blot analysis showed Dox treatment rapidly increased the expression of ERRγ (Figure 1F), but not ERRα (Figure S1G), in both HepG2 and MCF-7 cells. This might be due to that Dox treatment can increase the mRNA of ERRγ expression in cancer cells (Figure 1G). Collectively, these data showed that ERRγ is upregulated in chemoresistant cancer cells.

### ERRγ regulates chemoresistance of cancer cells

**Figure 2:**
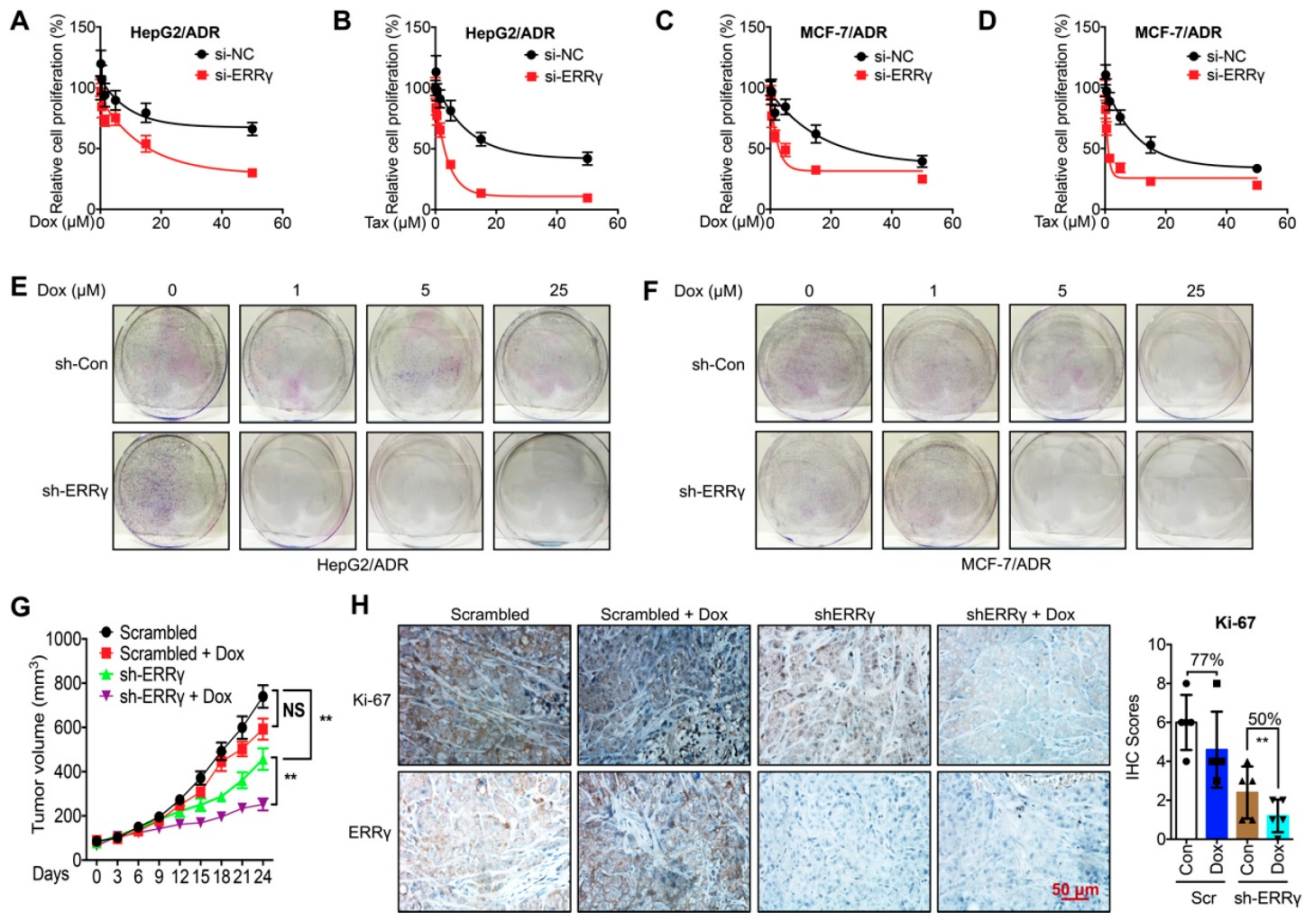
** ERRγ regulates chemoresistance of cancer cells.** (A&B) Cell proliferation rate in si-NC- or si-ERRγ-1-transfected HepG2/ADR cells for 24 h and followed by treatment with increasing concentrations of Dox (A) or Tax (B) for 48 h; (C&D) Cell proliferation rate in si-NC- or si-ERRγ-1-transfected MCF-7/ADR cells for 24 h and followed by treatment with increasing concentrations of Dox (C) or Tax (D) for 48 h; (E&F) HepG2/ADR (E) or MCF-7/ADR (F) cells transfected with scrambled shRNA or sh-ERRγ were split and cultured in fresh medium for the next 15 days. The colonies were fixed with methanol/glacial acetic acid (7:1) and stained with 0.1% of crystal violet; (G) Tumor volume measurement in mouse xenografts. HepG2/ADR cells stably transfected with scrambled shRNA or sh-ERRγ were subcutaneously inoculated in nude mice. We randomly divided the mice into Scramble, sh-ERRγ, Dox + Scramble and Dox + sh-ERRγ groups and treated them as described in the Methods. Tumor growth curves were constructed based on the tumor volumes measured in the mice; (H) IHC analysis of mouse xenograft tissues. Expression of ERRγ and proliferation marker Ki-67 was determined in tumor tissue sections from the xenografts using IHC (scale bar, 50 μm) and quantitatively analyzed; Data were presented as means ± SD from three independent experiments. **p< 0.01. NS, no significant.

To determine whether ERRγ is involved in chemoresistance of cancer cells, we knocked down the expression of ERRγ by siRNA transfection (Figure S2A). Knockdown of ERRγ significantly restored Dox and Tax sensitivity in HepG2/ADR cells, with IC_50_ values of Dox for si-NC and si-ERRγ being 173 and 15.9 μM (Figure 2A) and of Tax for si-NC and si-ERRγ being 29.2 and 2.36 μM, respectively (Figure 2B). As to MCF-7/ADR cells, the IC_50_ values of Dox for si-NC and si-ERRγ were 30.2 and 4.96 μM (Figure 2C) and of Tax for si-NC and si-ERRγ were 23.4 and 2.76 μM, respectively (Figure 2D). Colony formation assay showed that knockdown of ERRγ significantly inhibited colonization of both HepG2/ADR (Figure 2E) and MCF-7/ADR (Figure 2F) cells. In contrast, overexpression of ERRγ in HepG2 and MCF-7 cells by transfection with pcDNA/ERRγ (Figure S2B) decreased the sensitivity of both cells to the treatment of Dox and Tax (Figure S2 C-F).

To evaluate whether ERRγ is essential for *in vivo* chemoresistance of cancer cells, we established mouse xenograft tumors by using sh-ERRγ-transfected HepG2/ADR cells (Figure S2G). The tumor growth rate and tumor size at the end of the experiments were significantly decreased in the sh-ERRγ group relative to the scrambled group (Figure 2G). When treated with Dox, the scrambled group showed no obvious decrease in tumor size as compared with that of the control group. As expected, sh-ERRγ increased* in vivo* Dox sensitivity of HepG2/Dox cells (Figure 2G). Subsequent IHC analysis confirmed the *in vivo* knockdown efficiency of sh-ERRγ (Figure 2H). Further, Dox treatment obviously reduced the expression of the proliferation marker Ki-67 in the sh-ERRγ group (50%) than in the scrambled group (77%) (Figure 2H). These data suggested that ERRγ regulates both *in vitro* and *in vivo* chemoresistance of cancer cells.

### P-gp is involved in ERRγ-regulated chemoresistance of cancer cells

**Figure 3:**
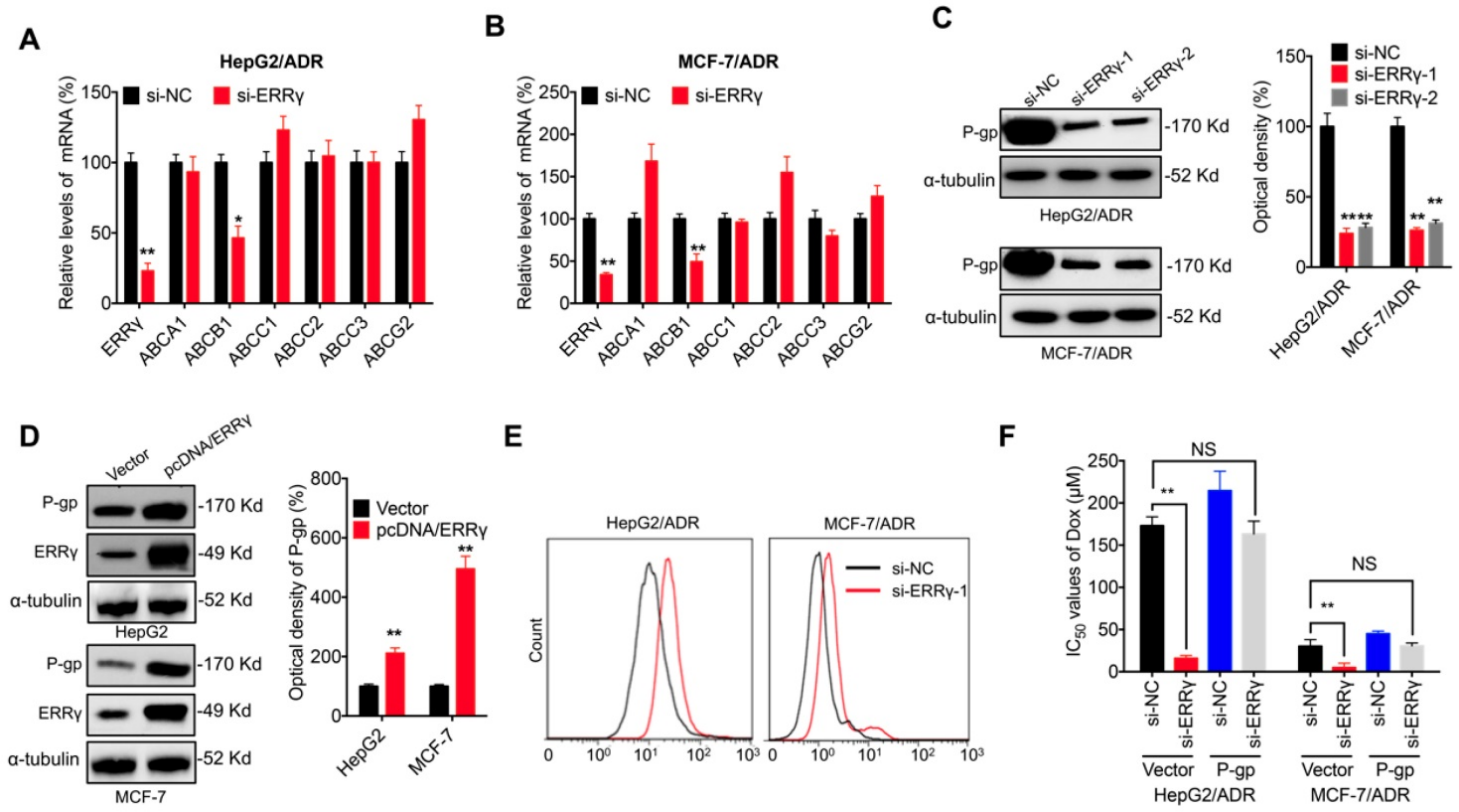
** P-gp is involved in ERRγ-regulated chemoresistance of cancer cells.** (A&B) mRNA expression of ABC transporters measured in HepG2/ADR (A) or MCF-7/ADR (B) cells 24 h post transfection with si-NC or si-ERRγ-1; (C) Expression of P-gp protein measured by Western blot analysis (left) and quantitively analyzed (right) in HepG2/ADR or MCF-7/ADR cells 24 h post transfection with si-NC or si-ERRγ-1/2; (D) Expression of P-gp protein measured by Western blot analysis (left) and quantitively analyzed (right) in HepG2 or MCF-7 cells 24 h post transfection with vector control or pcDNA/ERRγ; (E) P-gp function analyzed by flow cytometric measurement of the intracellular accumulation of Rh123 in HepG2/ADR or MCF-7/ADR cells 24 h post transfection with scrambled siRNA or si-ERRγ-1; (F) IC_50_ values of Dox in HepG2/ADR or MCF-7/ADR cells co-transfected with si-ERRγ and P-gp construct. Data were presented as means ± SD from three independent experiments. *p< 0.05, **p< 0.01. NS, no significant.

ABC transporters are critical for chemoresistance of cancer cells 6. Expression of the major ABC transporters, including *ABCA1*, *ABCB1*, *ABCC1*, *ABCC2*, *ABCC3* and *ABCG2,* was assessed in chemoresistant cancer cells transfected with si-ERRγ. qRT-PCR showed that si-ERRγ significantly decreased the expression of *ABCB1*, but not others, in both HepG2/ADR (Figure 3A) and MCF-7/ADR (Figure 3B) cells. Both si-ERRγ-1 and si-ERRγ-2 decreased *ABCB1* mRNA levels in MCF-7/Tax and MDA-MB-231/Tax cells (Figure S3A). Decreased protein expression of P-gp (encoded by *ABCB1*) was observed in both HepG2/ADR and MCF-7/ADR cells transfected with si-ERRγ-1 and si- ERRγ-2 (Figure 3C), while overexpression of ERRγ increased the expression of P-gp in both HpeG2 and MCF-7 cells (Figure 3D).

Although P-gp is known to mediate tumor cell chemoresistance 6, its role in ERRγ-regulated chemosensitivity was further investigated. Our data confirmed that si-ERRγ increased cellular accumulation of Rh123, a well-known fluorescent P-gp substrate, in both HepG2/ADR and MCF-7/ADR cells (Figure 3E). Overexpression of P-gp (Figure S3B) restored si-ERRγ-induced upregulation of Dox sensitivity in both HepG2/ADR and MCF-7/ADR cells (Figure 3F). These data indicated that P-gp is indeed involved in ERRγ-regulated chemoresistance of cancer cells.

### ERRγ interacts with p65 to regulate the transcription of *ABCB1*

**Figure 4:**
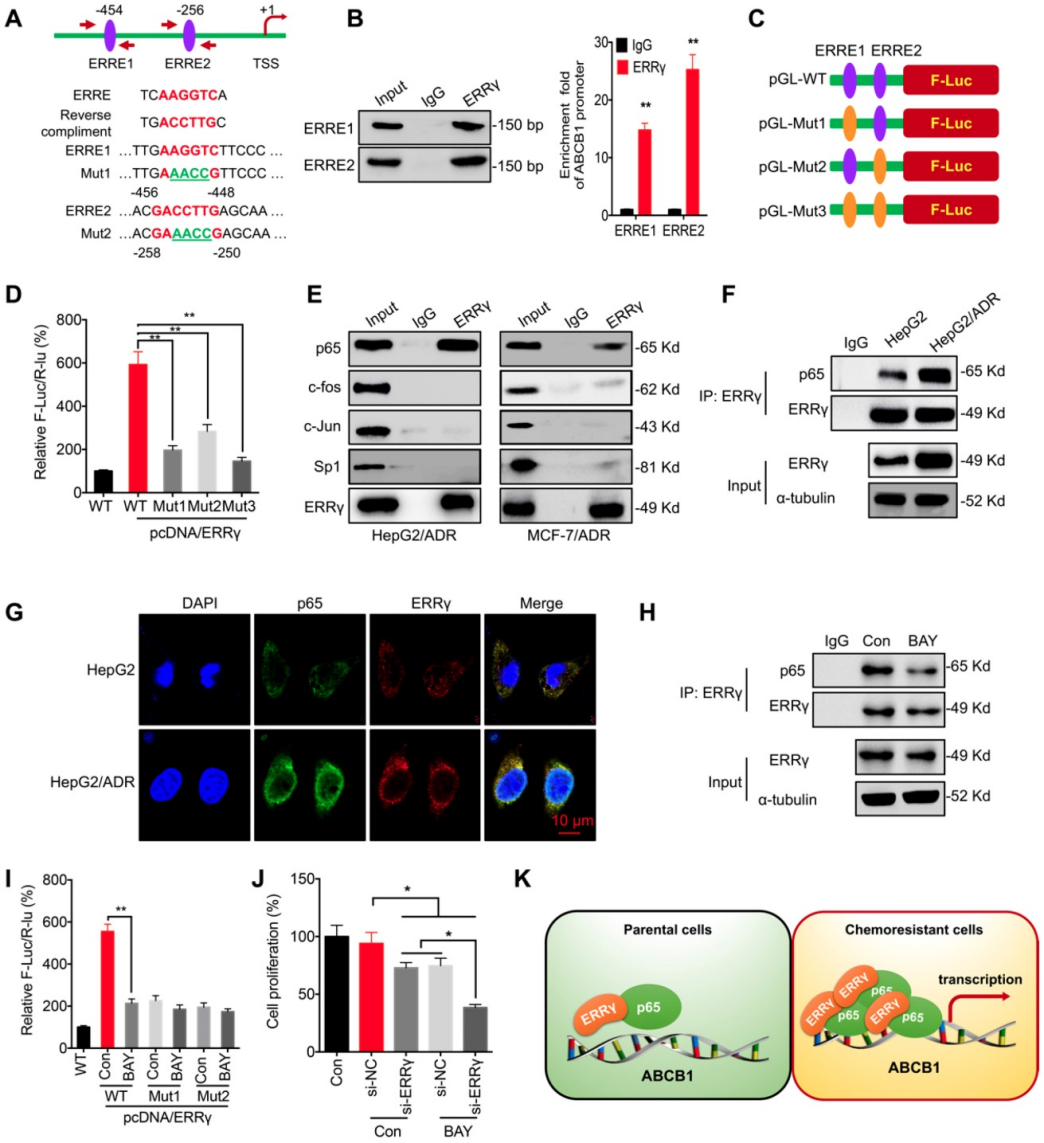
** ERRγ interacts with p65 to regulate *ABCB1* transcription.** (A) Schematic representation of ERREs in the promoter region of *ABCB1* with changes of nucleotides in ERRE1 and ERRE2 shown as indicated; (B) ChIP-PCR assay showing ERRγ binding to ERRE1 and ERRE2 in *ABCB1* promoter. The input (5%), binding between ERRγ and the promoter of *ABCB1* at the potential binding site ERRE1/2, was amplified by qPCR (*right*) and confirmed by 2% agarose gel electrophoresis (*left*); (C) Schematic representation of mutated ERRE positions in pGL-*ABCB1* vector; (D) Reporter gene assay performed in HepG2 cells 24 h post transfection with pGL-*ABCB1*-WT or pGL-*ABCB1*-Mut1/2/3 by dual-luciferase analysis; (E) Examination of ERRγ interaction with different transcription factors in HepG2/ADR and MCF-7/ADR cells following immunoprecipitation with ERRγ or control antibody and analyzed by Western blot analysis; (F) Interaction between ERRγ and p65 in HepG2 and HepG2/ADR cells monitored by immunoprecipitation using anti-ERRγ antibody; After ERRγ was immunoprecipitated, the binding between ERRγ and p65 was examined by Western blot analysis. An equal amount of ERRγ was loaded for normalization according to a pre-Western blot; (G) Expression and localization of p65 (*green*) and ERRγ (*red*) in HepG2 and HepG2/ADR cells visualized by confocal imaging; (H) Interaction between ERRγ and p65 in HepG2/ADR cells treated with or without BAY 11-7082 for 12 h and then analyzed by immunoprecipitation using an antibody against ERRγ; (I) Dual-luciferase reporter gene assay performed in HepG2 cells transfected with pGL-*ABCB1*-WT or pGL-*ABCB1*-Mut1/2, with or without pcDNA/ERRγ, for 12 h and then further treated with or without BAY 11-7082 for 12 h; (J) HepG2/ADR cells were treated with si-RNA or si-ERRγ combined with or without BAY 11-7082 for 12 h and then further treated with 5 μM Dox for 48 h; (K) Model for ERRγ/p65-promoted transcription of *ABCB1* in chemoresistant cancer cells. Data were presented as means ± SD from three independent experiments. **p< 0.01. NS, no significant.

The mechanism responsible for ERRγ-regulated transcription of *ABCB1* was further investigated. Computer-assisted searches of potential ERRγ-binding sites (ERR response element, ERRE, TNAAGGTCA) within the *ABCB1* promoter region (-1 kb) was conducted by using the TESS database, which predicts transcription factor-binding sites. Two putative ERREs located at -454 and -256 bp upstream of the transcription start site of the *ABCB1* promoter were identified (Figure 4A). ChIP-PCR confirmed that ERRγ binds to these two ERREs (Figure 4B). Next, an *ABCB1* promoter (-1024 to -1)-driven luciferase reporter assay was conducted with reporters containing wild-type (WT) or mutated (Mut) ERRγ-binding sites (Figure 4A and 4C). As shown in Figure 4D, ERRγ induced robust luciferase expression in pGL3-*ABCB1*-WT, whereas mutation at ERRE1 and/or ERRE2 significantly decreased ERRγ-induced luciferase activity, suggesting that ERRγ binds directly to the *ABCB1* promoter to regulate its transcription.

ERRγ can form homodimers and heterodimers via its ligand-binding domain (LBD) 21, 22. We hypothesized that transcription factors regulating *ABCB1* expression, including c-Jun, c-Fos, NF-κB/p65, and Sp1 23, 24, might interact with ERRγ to increase its activity. Co-IP showed that ERRγ associated with endogenous p65, but not the other transcription factors, in HepG2/ADR and MCF-7/ADR cells (Figure 4E). Consistently, reciprocal co-IP showed that ERRγ was pulled down in HepG2/ADR and MCF-7/ADR cell lysates by anti-p65 antibody (Figure S4A). To compare the binding between ERRγ and p65 in chemoresistant and parental cells, an equal amount of ERRγ after immunoprecipitation by use of its antibody was loaded for normalization according to a pre-Western blot since the endogenous ERRγ was increased in HepG2/ADR cells. The data showed that the binding between ERRγ and p65 was increased in HepG2/ADR cells as compared with that in HepG2 cells (Figure 4F). Confocal imaging showed enhanced expression of ERRγ in HepG2/ADR cells and colocalized with p65 in both HepG2 and HepG2/ADR cells (Figure 4G). These results suggest that the interaction between ERRγ and p65 was upregulated in chemoresistant cells.

We further investigated whether p65 was involved in ERRγ-regulated transcription of *ABCB1*. An inhibitor of NF-κB, BAY 11-7082, suppressed the mRNA (Figure S4B) and protein (Figure S4C) expression of P-gp in HepG2/ADR and MCF-7/ADR cells. BAY 11-7082 also decreased the interaction between p65 and ERRγ in HepG2/ADR cells (Figure 4H). Moreover, BAY 11-7082 could decrease the promoter activity of pGL3-*ABCB1*-WT, while had no significant effect on the relative F-Luc/R-Luc for pGL-*ABCB1*-Mut-1/2 (Figure 4I), suggesting that ERRγ was involved in p65-regulated transcription of *ABCB1*. Further, our data showed that BAY 11-708 can significantly elevate the si-ERRγ-increased Dox sensitivity of HepG2/ADR cells (Figure 4 J). Our data suggest that ERRγ can interact with p65 to promote *ABCB1* transcription in chemoresistant cells (Figure 4K).

### ERRγ dictates metabolic reprogramming in chemoresistant cancer cells

**Figure 5:**
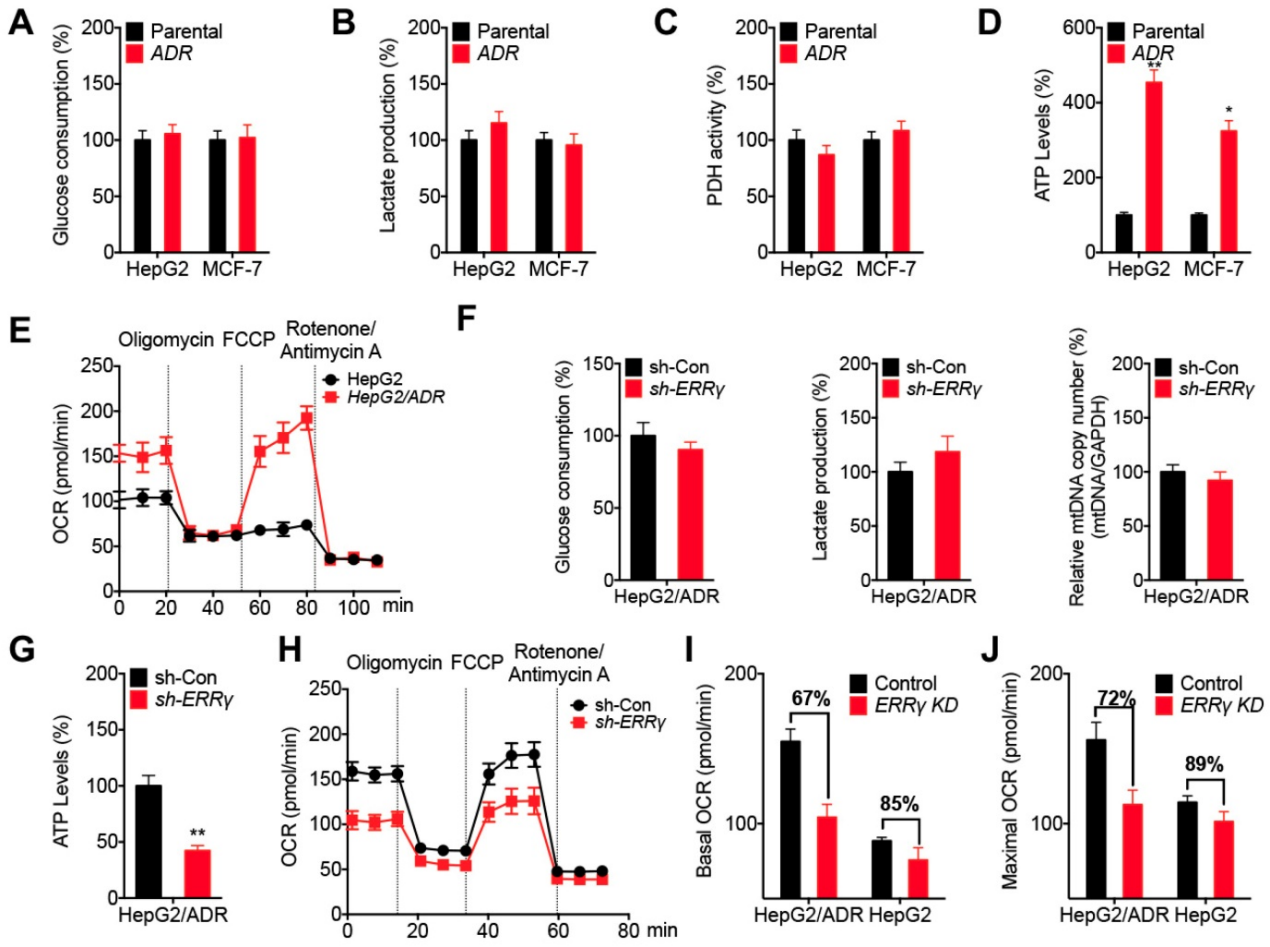
** ERRγ dictates the metabolic reprogramming in chemoresistant cancer cells.** (A~D) Relative glucose consumption (A), lactate production (B), PDH activity (C), and ATP levels (D) measured in HepG2/ADR or MCF-7/ADR cells following comparison with those measured in their corresponding parental cells; (E) OCR in HepG2/ADR and HepG2 cells measured by Seahorse XF24 analyzer; (F) Relative glucose consumption, lactate production, and mitochondrial mass in HepG2/ADR cells transfected with sh-Con or sh-ERRγ; (G) Relative ATP levels in HepG2/ADR cells transfected with sh-Con or sh-ERRγ; (H) OCR in HepG2/ADR cells transfected with sh-Con or sh-ERRγ measured by Seahorse XF24 analyzer; (I~J) Basal (I) and maximal (J) OCR measured by Seahorse XF24 analyzer in HepG2/ADR or HepG2 cells transfected with sh-Con or sh-ERRγ. Data were presented as means ± SD from three independent experiments. *p< 0.05, **p< 0.01.

Chemoresistant cancer cells diverge metabolic properties such as aerobic glycolysis and mitochondrial respiration 25, 26. Our data showed that HepG2/ADR and MCF-7/ADR cells exhibited no significant difference in glucose consumption (Figure 5A) and lactate production (Figure 5B) rates compared to that of their corresponding parental cells. Further, the activity of pyruvate dehydrogenase (PDH), which converts pyruvate to acetyl-CoA (Ac-CoA) and then enters the TCA cycle 27, did not vary between chemoresistant and parental cells (Figure 5C). However, the extracellular ATP levels in chemoresistant cells were significantly greater than that in the parental cells (Figure 5D). Seahorse analysis showed that HepG2/ADR cells displayed an increased basal and maximal oxygen consumption rate (OCR), an indicator of mitochondrial oxidative respiration (Figure 5E), but had comparable levels of the extracellular acidification rate (ECAR), which reflects the overall glycolytic flux (Figure S5A), than that of HepG2 cells. However, the mitochondrial mass between HepG2/ADR and HepG2 cells had no significant difference (Figure S5B). These data suggested that chemoresistant cells showed increased ATP production and OCR than that of parental cells.

We further investigated the potential roles of ERRγ in metabolic programming of chemoresistant cancer cells. Our data showed that knockdown of ERRγ had no significant effect on glucose consumption, lactate production, or mitochondrial mass in HepG2/ADR cells (Figure 5F). However, knockdown of ERRγ decreased the ATP levels of HepG2/ADR cells (Figure 5G) as well as the basal and maximum OCRs (Figure 5H), but had no significant effect on ECAR (Figure S5C) in HepG2/ADR cells. This was further confirmed by overexpression of ERRγ resulting in increased basal and maximum OCRs without significantly altering ECAR in HepG2 cells (Figure S5D and S5E). Moreover, knockdown of ERRγ more effectively decreased the basal (Figure 5I) and maximum (Figure 5J) OCRs of HepG2/ADR cells than that in HepG2 cells. Both overexpression of ERRγ in HepG2 cells (Figure S5F) and knockdown of ERRγ in HepG2/ADR cells (Figure S5G) had no significant effect on key gene expression involved in oxidative phosphorylation (OxPhos) such as *CS, NDUFA4, SDHB, COX5B,* or *ATP5B,* suggesting that ERRγ-upregulated OCR and ATP production were not related to the OxPhos pathway. All the data indicated that ERRγ dictates metabolic reprogramming in chemoresistant cancer cells without affecting glycolysis, mitochondrial mass, and PDH activities, but it does increase ATP generation and OCR.

### ERRγ regulates FAO via Cpt1b in chemoresistant cancer cells

**Figure 6:**
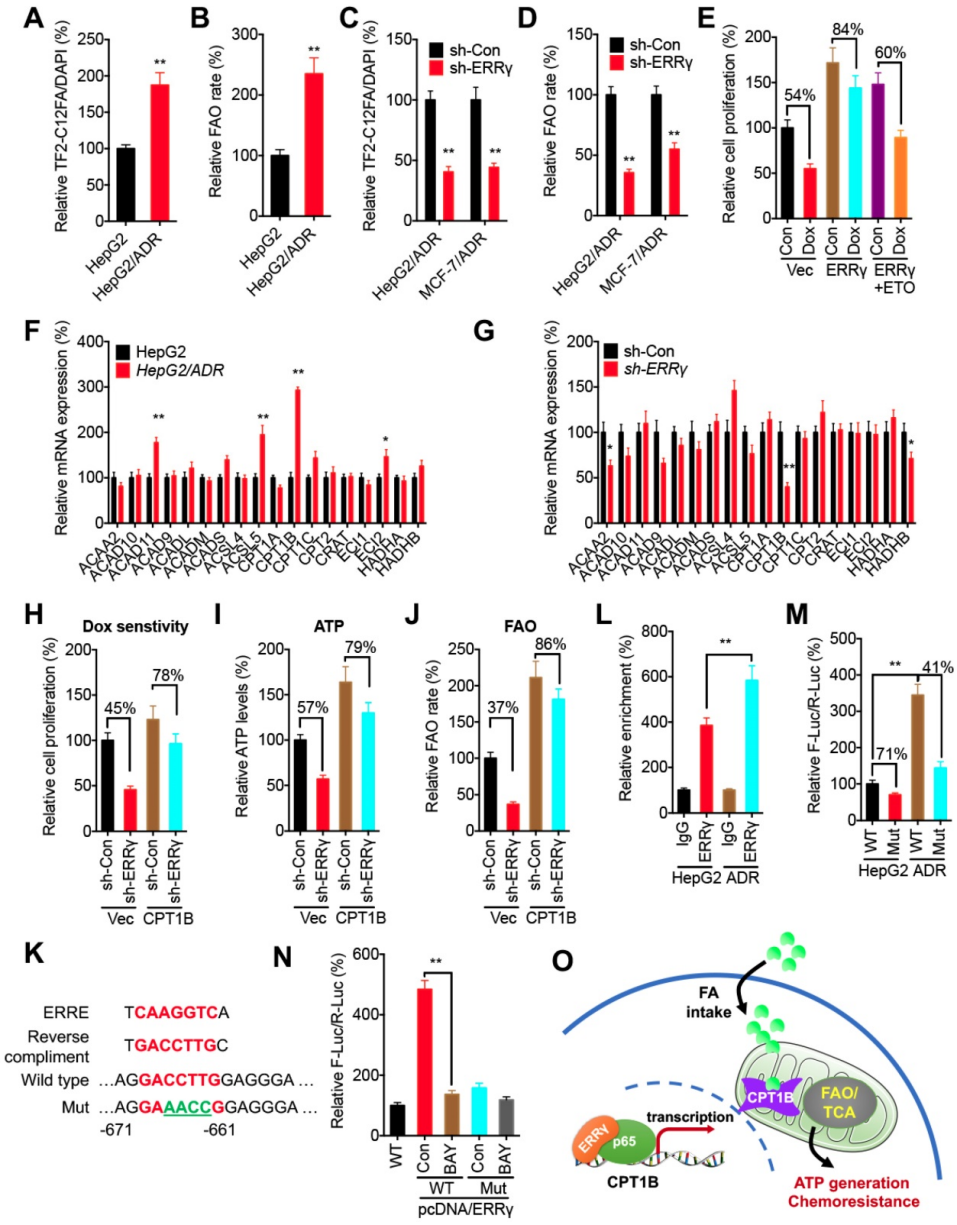
** ERRγ regulates the FAO via Cpt1b in chemoresistant cancer cells.** (A~B) Relative FA uptake (A) and FA β oxidation rate (B) between HepG2 and HepG2/ADR cells; (C~D) Relative FA uptake (C) and FA β oxidation rate (D) in cells transfected with sh-Con or sh-ERRγ; (E) Cell proliferation measured by CCK-8 kit in HepG2 cells pre-transfected with vector control or pcDNA/ERRγ for 6 h and then treated with or without Dox (1 μM) combined with or without ETO for 24 h; (F) mRNA levels of FAO-related genes measured by qRT-PCR in HepG2 and HepG2/ADR cells; (G) mRNA levels of FAO-related genes measured by qRT-PCR in HepG2/ADR cells transfected with sh-Con or sh-ERRγ; (H) Cell proliferation measured by CCK-8 kit in HepG2/ADR cells pre-transfected with sh-Con or sh-ERRγ and then transfected with vector or a Cpt1b expression construct, followed by further treatment with Dox (10 μM) for 24 h; (I~J) ATP (I) and FA β oxidation rate (J) measured in HepG2/ADR cells transfected with sh-Con or sh-ERRγ with further transfection with vector or a Cpt1b expression construct for 24 h; (K) Nucleotide sequences of ERREs in *CPT1B* and the mutated (GACCTTG to AGAACCG) nucleotides in pGL3-*CPT1B*-Mut-Luc vector; (L) ChIP assay measuring ERRγ binding to *CPT1B* promoter in both HepG2 and HepG2/ADR cells; (M) Dual-luciferase reporter gene assay performed in HepG2 and HepG2/ADR cells transfected with pGL3-*CPT1B*-WT-Luc or pGL3-*CPT1B*-Mut-Luc; (N) Dual-luciferase reporter gene assay performed in HepG2 cells transfected with pGL-*ABCB1*-WT or pGL3-*CPT1B*-Mut reporter with or without pcDNA/ERRγ for 12 h and then further treated with or without BAY 11-7082 for 12 h; (O) Model for ERRγ-regulated FAO via Cpt1b in chemoresistant cancer cells. Data were presented as means ± SD from three independent experiments. *p< 0.05.

A recent study indicates that ERRγ in kidney cells can regulate mitochondrial FAO functions via direct binding to FAO genes such as *CPT1B, CPT2, ACADM,* and *HADHA* 28. We then investigated the potential effects of ERRγ on mitochondrial FAO functions in cancer cells. We found the FA uptake (Figure 6A) and FAO rate (Figure 6B) in HepG2/ADR cells were significantly greater than that in HepG2 cells. Knockdown of ERRγ significantly inhibited the FA uptake (Figure 6C) and FAO rate (Figure 6D) in both HepG2/ADR and MCF-7/ADR cells. Further, overexpression of ERRγ decreased Dox sensitivity of HepG2 cells, while this effect could be blocked by the FAO inhibitor etomoxir (ETO) (Figure 6E), suggesting that upregulation of FAO was involved in ERRγ-induced chemoresistance of cancer cells.

To investigate the mechanisms of ERRγ-regulated FAO in cancer cells, qRT-PCR was performed to evaluate the expression of genes involved in cellular FAO **(Table S1)** 29. Among all of the FAO-related genes examined, expression of *CPT1B* was significantly increased in HepG2/ADR cells as compared with that in HepG2 cells (Figure 6F). Knockdown performed with sh-ERRγ significantly decreased *CPT1B* expression in HepG2/ADR cells (Figure 6G). Consistently, overexpression of ERRγ increased the expression of *CPT1B* in HepG2 cells (Figure S6A), while both si-ERRγ-1 and si-ERRγ-2 significantly decreased the protein levels of Cpt1b in HepG2/ADR cells (Figure S6B). To investigate whether Cpt1b was involved in ERRγ-regulated FAO and chemoresistance, we overexpressed Cpt1b in HepG2/ADR cells transfected with sh-Con or sh-ERRγ (Figure S6C). Our data showed that overexpression of Cpt1b significantly attenuated sh-ERRγ-increased sensitivity of Dox in HepG2/ADR cells (Figure 6H), and also sh-ERRγ-downregulated ATP levels (Figure 6I) and FAO rate (Figure 6J), suggesting that Cpt1b is involved in ERRγ-regulated FAO and chemoresistance of cancer cells.

Cpt1b has been indicated as a direct transcriptional target of ERRγ 28, but the binding site(s) in the *CPT1B* promoter has not been well studied. Analysis of the region 1.0 kb upstream from the transcription start site in the *CPT1B* promoter revealed one putative ERRE, showing 78% homology (7/9) to the consensus ERRE (Figure 6K). ChIP assay confirmed that binding of ERRγ to *CPT1B* in HepG2/ADR cells was greater than that in HepG2 cells (Figure 6L). We then cloned the promoter of *CPT1B* to generate pGL3-*CPT1B*-WT-Luc and mutated the bind site (to GA**AACC**G) to generate pGL3-*CPT1B*-Mut-Luc (Figure 6K). The promoter activity of pGL3-*CPT1B*-WT-Luc in HepG2/ADR cells was significantly greater than that in HepG2 cells; however, pGL3-*CPT1B*-Mut-Luc attenuated this difference between HepG2/ADR and HepG2 cells (Figure 6M). In general, the promoter activity of pGL3-*CPT1B*-WT-Luc was greater than that of pGL3-*CPT1B*-Mut-Luc in both HepG2/ADR and HepG2 cells (Figure 6M).

We further investigated whether p65/ERRγ complex was involved in the upregulation of Cpt1b in chemoresistant cells. BAY 11-7082, the inhibitor of p65/NF-κB, suppressed the mRNA (Figure S6D) and protein (Figure S6E) levels of Cpt1b in both HepG2/ADR and MCF-7/ADR cells and also significantly decreased ERRγ-induced promoter activity of pGL3-*CPT1B*-WT-Luc, while it had less effect on the activity of pGL3-*CPT1B*-Mut-Luc in HepG2 cells (Figure 6N). Our data suggest that ERRγ forms a complex with p65, binds *CPT1B* promoter to increase its expression, elevates FAO, and thus mediate chemoresistance of cancer cells (Figure 6O).

### The m^6^A-facilitated splicing increases the expression of ERRγ

The potential epigenetic mechanisms responsible for the upregulation of ERRγ in chemoresistant cells were investigated. Firstly, treatment with 5-aza-dC (a DNA methyltransferase inhibitor) had no significant effect on ERRγ expression in either HepG2 or HepG2/ADR cells (Figure S7 A), suggesting that DNA methylation might not be involved in ERRγ expression. Further, broad-spectrum HDAC inhibitors including SAHA and NaB also had no obvious effect on the expression of ERRγ in HepG2 or HepG2/ADR cells (Figure S7B), indicating histone acetylation might not be responsible for the upregulation of ERRγ in chemoresistant cells.

**Figure 7:**
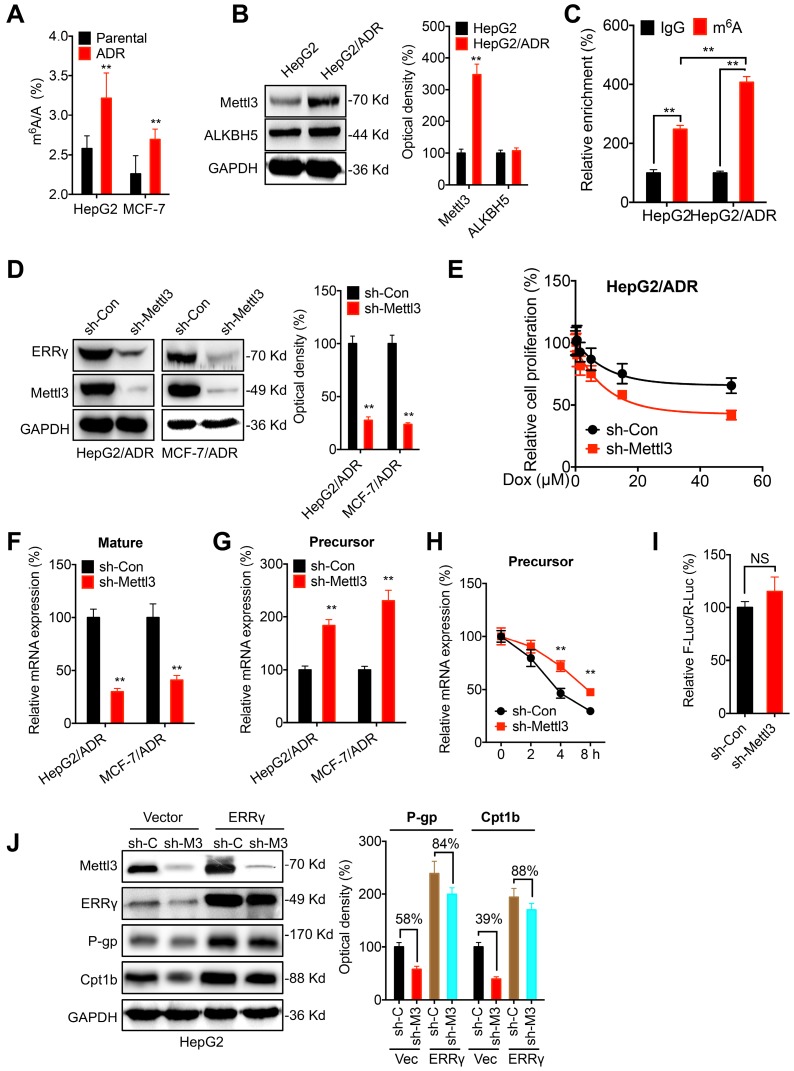
** The m^6^A-facilitated splicing is responsible for the upregulation of ERRγ.** (A) The m^6^A/A ratio of total mRNA in HepG2/ADR and MCF-7/ADR cells were determined by LC-MS/MS and compared with that in their parental cells; (B) The expression of Mettl3 and ALKBH5 in HepG2/ADR and HepG2 cells was checked by western blot analysis and quantitatively analyzed; (C) m^6^A RIP-qPCR analysis of ERRγ mRNA in HepG2/ADR and HepG2 cells; (D) The expression of ERRγ in HepG2/ADR and MCF-7/ADR cells transfected with sh-Con or sh-Mettl3 was checked by western blot analysis and quantitatively analyzed; (E) HepG2/ADR cells transfected with sh-Con or sh-Mettl3 were further treated with increasing concentrations of Dox, the cell proliferation was tested by CCK-8 kit; (F~G) The mature (E) and precursor (F) mRNA of ERRγ in HepG2/ADR cells transfected with sh-Con or sh-Mettl3 were checked by qRT-PCR; (H) HepG2/ADR cells transfected with sh-Con or sh-Mettl3 were pre-treated with Act-D for 90 min, then the precursor mRNA of ERRγ was checked by qRT-PCR; (I) The promoter activity of pGL-ESRRG-Basic in HepG2/ADR cells transfected with sh-Con or sh-Mettl3 was checked by dual luciferase assay; (J) HepG2 cells were transfected with sh-Con, sh-Mettl3, vector control or ERRγ construct alone or together for 24 h, the expression of targets was measured and quantitatively analyzed. Data were presented as means ± SD from three independent experiments. **p< 0.01. NS, no significant.

Recent investigations indicated that *N6*-methyladenosine (m^6^A) modification can regulate gene expression and be involved in chemoresistance of cancer cells 30, 31. Intriguingly, we found that the m^6^A of mRNA was increased in HepG2/ADR and MCF/ADR cells as compared to their parental cells (Figure 7 A). Western blot analysis showed that the expression of m^6^A methyltransferase Mettl3 was upregulated in HepG2/ADR cells, while the expression of demethylase ALKBH5 had no variation (Figure 7 B). m^6^A-RIP-qPCR confirmed that a 2-fold m^6^A antibody enriched *ESRRG* mRNA in HepG2 cells, while this enrichment was significantly increased in HepG2/ADR cells (Figure 7 C). We found that knockdown of Mettl3 can decrease the expression of ERRγ in both HepG2/ADR and MCF-7/ADR cells (Figure 7 D). Consistently, over expression of Mettl3 increased the expression of ERRγ in HepG2 cells (Figure S7 C). Further, knockdown of Metttl3 can significantly increase the Dox sensitivity of HepG2/ADR cells (Figure 7 E). It indicated that m^6^A can increase the expression of ERRγ in cancer cells.

We then investigated the potential mechanisms responsible for m^6^A regulated expression of ERRγ. Firstly, knockdown of Mettl3 had no significant effect on the protein stability of ERRγ in HepG2/ADR cells (Figure S7 D). However, knockdown of Mettl3 can significantly decrease mature mRNA of ERRγ (Figure 7 F). Consistently, over expression of Mettl3 can increase the mRNA expression of ERRγ in both HepG2 and MCF-7 cells (Figure S7 E). The Mettl3 induced upregulation might not be due to the nuclear turnover or mRNA degradation since knockdown of Mettl3 had no significant effect on either the subcellular localization of mature mRNA (Figure S7 F) or the mRNA stability (Figure S7 G). Intriguingly, we found that knockdown of Mettl3 can increase the precursor mRNA of ERRγ (Figure 7 G), which might be due to that knockdown of Mettl3 can increase the half-life of precursor mRNA (Figure 7 H). It suggested that the decrease of m^6^A can delay the splicing of precursor mRNA of ERRγ to suppress its expression. This was confirmed by the promoter activity assay which showed that knockdown of Mettl3 had no significant effect on the pGL-ESRRG-Basic (Figure 7 I), suggesting that m^6^A had no effect on the transcription of ERRγ. Further, we found that in HepG2 cells, knockdown of Mettl3 resulted in the down regulation of P-gp and Cpt1b, however, over expression of ERRγ can significantly attenuate knockdown of Mettl3 induced down regulation of P-gp and Cpt1b (Figure 7 J). Collectively, these data suggested that m^6^A can trigger the splicing of ERRγ precursor mRNA and then regulate the phenotype of chemoresistance.

### The m^6^A/ERRγ axis and *in vivo* cancer progression

**Figure 8:**
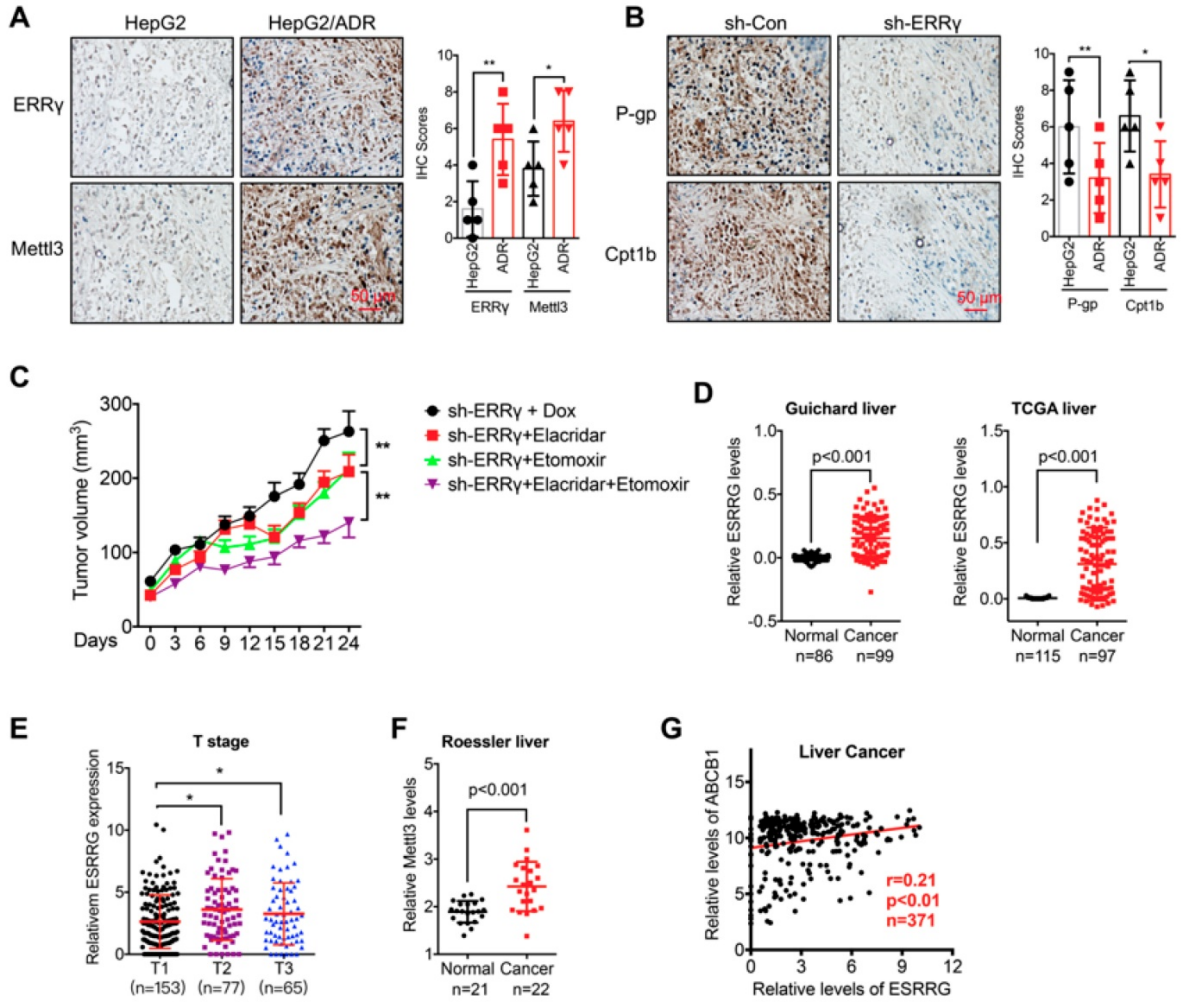
** The m^6^A/ ERRγ axis and *in vivo* cancer progression.** (A) IHC (ERRγ and Mettl3)-stained paraffin-embedded sections obtained from HepG2 and HepG2/ADR xenografts when the tumor volumes were about 100 mm^3^ for each group; (B) The sh-control and sh-Mettl3 HepG2/ADR cells were subcutaneously inoculated in nude mice. IHC (P-gp and Cpt1b)-stained paraffin-embedded sections obtained at the end of experiment; (C) Tumor volume measurement in mouse xenografts. HepG2/ADR cells stably transfected with sh-ERRγ were subcutaneously inoculated in nude mice. We randomly divided the mice into sh-ERRγ, sh-ERRγ + Elacridar, sh-ERRγ + Etomoxir, and sh-ERRγ + Elacridar + Etomoxir and then treated with Dox as described in the Methods. Tumor growth curves were constructed based on the tumor volumes measured in the mice; (D) Expression of ESRRG in HCC tumor tissues and normal liver tissues from Oncomine database (Guichard and TCGA liver cancers); (E) ESRRG expression in liver cancers of T1 (n=153), T2 (n=77), and T3 (n=65) stages from TCGA database; (F) Expression of Mettl3 in HCC tumor tissues and normal liver tissues from Oncomine database (Roessler liver); (G) Correlation between *ESRRG* and *ABCB1* in liver cancer patients (n=371) from TCGA database; Data were presented as means ± SD from three independent experiments. *p<0.05, **p< 0.01.

To define the molecular basis of the ERRγ in cancer progression, we checked the expression of Mettl3 and ERRγ in xenografts based on HepG2 and HepG2/ADR cells. Our data showed that when the tumor volume was comparable, the expression of ERRγ and Mettl3 was increased in HepG2/ADR groups as compared to that in parental cells (Figure 8 A). We further checked the *in vivo* effects of sh-ERRγ on the expression of P-gp and Cpt1b in chemoresistance cells. Our data showed that knockdown of ERRγ can further decrease the expression of P-gp and Cpt1b in HepG2/ADR xenograft model (Figure 8 B). These data indicated that m^6^A/ ERRγ/P-gp-Cpt1b axis was involved in the *in vivo* progression and chemoresistance of cancer cells. Further, we established mouse xenograft tumors by using sh-ERRγ-transfected HepG2/ADR cells. Then the xenograft tumors were then treated with Dox combined with elacridar (P-gp inhibitor) 32 or etomoxir (Cpt1 inhibitor) 8, our data showed that the combination of elacridar or etomoxir can increase the *in vivo* Dox sensitivity of HepG2/Dox cells. Further, the combination of elacridar and etomoxir had synergistic effects to increase the sh-ERRγ-restored *in vivo* Dox sensitivity of HepG2/Dox cells (Figure 8C). It confirmed that ERRγ can regulate the *in vivo* chemoresistance via modulation of P-gp and Cpt1b.

We then analyzed the expression of m^6^A/ERRγ axis and their correlation with clinical characteristics of breast and liver cancers. Increased expression of ERRγ in liver cancer versus normal tissue has been observed in Guichard and TCGA data from Oncomine database (Figure 8D). Consistently, increased expression of ERRγ was also observed in breast cancer versus normal tissues in Finak data from Oncomine database (Figure S8 A). Further, significant rising expression levels of *ESRRG* from T1 to T3 stage of liver cancer tissues were observed (Figure 8 E), implying an increasing tendency of *ESRRG* expression during malignant transformation.

In addition, increased expression of Mettl3 has also be found in Roessler liver cancer (Figure 8 F). Further, we assessed the correlation between ERRγ and P-gp in cancer patients with data downloaded from LinkedOmics (http://www.linkedomics.org). Our data showed that the expression of ERRγ was positively correlated with the ABCB1 in liver (Figure 8 G) and breast (Figure S8 B) cancer patients. All these data suggested that ERRγ regulated chemoresistance and metabolic reprogramming might be involved in the *in vivo* cancer progression.

## Discussion

Previous studies indicated that ERR signaling can regulate the progression of various cancers 19, 33, 34. Our present study found that ERRγ was upregulated in chemoresistant cancer cells and targeted inhibition of ERRγ restored the chemosensitivity. Mechanistically, ERRγ can interact with p65 and bind to the promoter of *ABCB1*, which encodes a key transporter that pumps many foreign substances out of cells, to increase its transcription and expression. At the same time, ERRγ can facilitate FAO of chemoresistant cancer cells via upregulation of Cpt1b. Intriguingly, chemoresistant cells showed increased levels of m^6^A and expression of Mettl3 than that of parental cells, which can trigger the splicing of precursor of *ESRRG* mRNA to increase it expression. Collectively, we found that m^6^A-induced ERRγ is essential for chemoresistance of cancer cells through upregulation of *ABCB1* and metabolic reprogramming.

Since ERRγ is upregulated in resistant cells, targeted inhibition of ERRγ can increase *in vitro* and *in vivo* sensitivity to chemotherapy. In hormone therapy of breast cancer cells, ERRγ is upregulated during the acquisition of TAM resistance in estrogen receptor-positive (ER+) breast cancer cells, thus overexpression of ERRγ is sufficient to induce TAM resistance 18 via cooperation with proline, glutamic acid and leucine rich protein 1 (PELP1) to inhibit TAM-mediated cell death 35. ERRγ target genes are poor prognostic factors in TAM-treated breast cancer 36. As to cancer progression, the role of ERRγ seems to be paradoxical. ERRγ acts as a tumor suppressor in gastric cancer by directly targeting the Wnt signaling pathway 37. Conversely, ERRγ is upregulated in liver cancer and exerts oncogenic potential by suppressing *p21* and *p27* 38. The diverse roles of ERRγ is likely dependent on cell context and its functional interactions with cell-specific transcription factors and co-regulators. Our present study revealed that essential roles of ERRγ in chemoresistance of cancer cells. Nowadays, GSK5182, the inverse agonist of ERRγ, has been reported to inhibit the biological functions of ERRγ in cardiac hypertrophy 39, iron homeostasis 40, and cancer cell proliferation 41. The therapeutic potential of GSK5182 on cancer chemoresistance needs further investigations.

Continuing our finding that ERRγ interacts with p65 to trigger the transcription and expression of *ABCB1,* we identified two ERREs in the promoter region of *ABCB1* and proved that both ERREs are involved in ERRγ-regulated expression of P-gp. NF-κB appears to play a dual role in the regulation of *ABCB1* 42. It can bind to -167 and -158 of the *ABCB1* promoter to activate its transcription in liver cancer cells 43. In our study, the inhibitor (BAY) of NF-κB can attenuate ERRγ-induced transcription of *ABCB1*, potentially attributed to BAY-abrogated NF-κB binding to DNA 44 and thus reducing ERRγ/p65 association with the *ABCB1* promoter. Our results also revealed an essential role of NF-κB in ERRγ-induced expression of *ABCB1* and chemoresistance, which is further supported by clinical data confirming that expression of *ABCB1* and ERRγ was positively correlated in liver cancer tissues.

Metabolic reprogramming is one of the hallmarks for cancer cell growth and progression, as well as resistance to chemotherapy 45. We found that chemoresistant cells reprogram metabolic pathways without affecting glycolysis, mitochondrial mass, and PDH activities but increasing ATP generation and OCR through acceleration of FAO. Recent evidence underscores the idea that FAO, also called β-oxidation, is an important energy resource required for cancer cell growth, survival, and metastasis 13, 46. Inhibition of FAO is identified as a new therapeutic approach for MYC-overexpressing triple-negative breast cancer (TNBC) 8. Some studies indicate that FAO is able to support breast cancer stem cell self-renewal and is a characteristic of chemoresistant cancer cells 10. The upregulation of FAO may thus confer the chemoresistance through maximizing ATP production, decrease intracellular ROS, and eventually protect cancer cells from death 13, 47. Our data confirmed that targeted FAO might be helpful in overcoming chemoresistance of cancer cells.

According to our data, ERRγ-regulated Cpt1b was responsible for facilitated FAO in chemoresistant cancer cells. ERRγ plays an important role in metabolism to promote energy-generating mitochondrial functions in several energy-demanding cell types 17. Genomic studies revealed that ERRγ binds directly to and activates the transcription of hundreds of genes involved in mitochondrial OxPhos/FAO functions, including *CPT1B, CPT2, ACADM,* and *HADHA* 28, 48. Consistently, we identified the ERRE located at -671 to -661 of the *CPT1B* promoter is involved in ERRγ-regulated transcription and expression. Cpt1b, which is responsible for fatty acid transport into mitochondria for β-oxidation, localizes at the outer mitochondrial membrane and acts as the rate-limiting FAO enzyme 49. We found that overexpression of Cpt1b can reverse sh-ERRγ-sensitized Dox treatment and downregulation of ATP and FAO, while elevated Cpt1b expression correlates with poorer response to chemotherapy 10. Considering ERRγ cistromes may exhibit cell-type-specific features to match the metabolic profiles of individual cell types 17, the roles of ERRγ/Cpt1b axis-regulated metabolic reprogramming in chemoresistance of other cancers will need more studies.

Intriguingly, we found that m^6^A was upregulated in chemoresistant cells and facilitated the splicing of precursor of *ERSSG* mRNA to elevate its expression. The roles of mRNA modification in controlling the cancer progression have just begun to be studied. Our recent study indicated that m^6^A can trigger the epithelial to mesenchymal transition of cancer cells via triggering the translation of Snail 50. In the present study, we showed that knockdown of Mettl3 can restore the chemosensitivity of HepG2/ADR cells, which is consistent with recent study that Mettl3 can promote the chemo- and radioresistance of pancreatic cancer cells 51. m^6^A can regulate the all stages in the life cycle of RNA including RNA processing, nuclear export and translation modulation 31, 52, 53. It has been reported that splicing regulators and m^6^A “reader” HNRNPC can regulate the splicing of target mRNAs in a m^6^A switch regulated manner 54. Whether HNRNPC is involved in Mettl3 and chemoresistance triggered splicing of ESRRG precursor mRNA need further studies.

In conclusion, we identified a key role of ERRγ in chemoresistance of cancer cells via upregulation of *ABCB1* and facilitation of FAO. We further uncovered novel mechanisms for ERRγ regulated transcription of ABCB1, revealed that Cpt1b mediated FAO is essential for chemoresistance, and found that m^6^A can trigger the cleave of precursor mRNA of *ESRRG* to decrease chemosensitivity. Our results provided a potent target that may serve as a predictive marker of chemotherapy and as an effective target for overcome chemoresistance.

## Supplementary Material

Supplementary figures, tables, materials and methods.

## Abbreviations

ABC: ATP-binding cassette
ABCB1: ATP binding cassette subfamily B member 1
Dox: doxorubicin
ECAR: extracellular acidification rate
ER: estrogen receptor
ERRγ: estrogen related receptor γ
ERRE: ERR response elements
ETX: etomoxir
FA: fatty acid
FAO: fatty acid oxidation
LBD: ligand-binding domain
m^6^A: N6-methyladenosine
MDR: multidrug resistance
TAM: tamoxifen
TCA: tricarboxylic acid cycle
TNBC: triple-negative breast cancer
OCR: oxygen consumption rate
OxPhos: oxidative phosphorylation
P-gp: P-glycoprotein
PDH: pyruvate dehydrogenase
PELP1: proline, glutamic acid and leucine rich protein 1
Tax: taxol
WT: wide-type
