# *N6*-methyladenosine-induced ERRγ triggers chemoresistance of cancer cells through upregulation of ABCB1 and metabolic reprogramming: Erratum

**DOI:** 10.7150/thno.85930

**Published:** 2023-07-06

**Authors:** Zhuojia Chen, Long Wu, Jiawang Zhou, Xinyao Lin, Yanxi Peng, Lichen Ge, Cheng-Ming Chiang, Hui Huang, Hongsheng Wang, Weiling He

**Affiliations:** 1 Guangdong Key Laboratory of Chiral Molecule and Drug Discovery, School of Pharmaceutical Sciences, Sun Yat-sen University, Guangzhou, Guangdong 510006, China; 2 Sun Yat-sen University Cancer Center; State Key Laboratory of Oncology in South China; Collaborative Innovation Center for Cancer Medicine, Guangzhou 510060, China; 3 Institute of Human Virology, University of Maryland School of Medicine, Baltimore, MD 21201, USA; 4 Department of Clinical Laboratory, Jinling Hospital, Nanjing University School of Medicine, 305 East Zhongshan Road, Nanjing 210002, China; 5 Simmons Comprehensive Cancer Center, Department of Pharmacology, and Department of Biochemistry, University of Texas Southwestern Medical Center, 5323 Harry Hines Boulevard, Dallas, Texas 75390, USA; 6 Cardiovascular Department, The Eighth Affiliated Hospital, Sun Yat-sen University, Shennan Middle Road 3025#, Shenzhen, 518033, China; 7 Department of Gastrointestinal Surgery, The First Affiliated Hospital, Sun Yat-sen University, Guangzhou, Guangdong, 510080, China

The authors regret to inform the readers that wrong selections of the relative insets may have occurred for Figure 1 D and Figure 7 D. The analyses have been performed on n=3 samples for each concentration and are not questionable. In order to reduce the misunderstanding that may be caused in the future, we replaced the images and bands with the alternative ones. It is now correct in the below pictures.

The authors would like to apologise for any inconvenience caused.

## Figures and Tables

**Figure 1:**
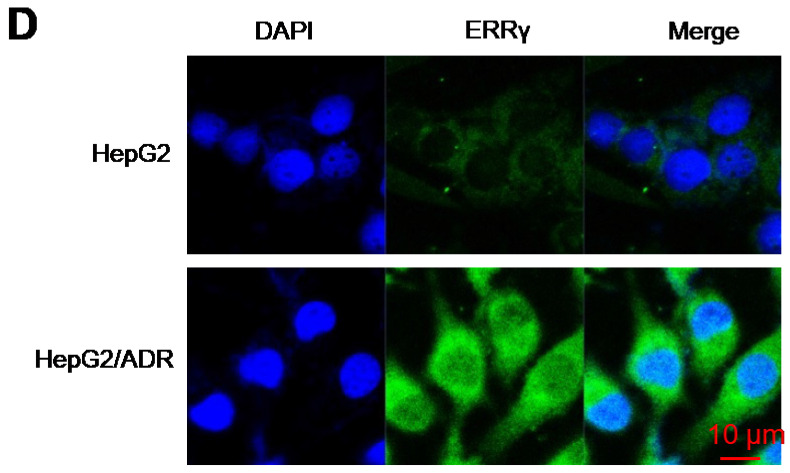
Corrected figure for the original Figure 1 D.

**Figure 7:**
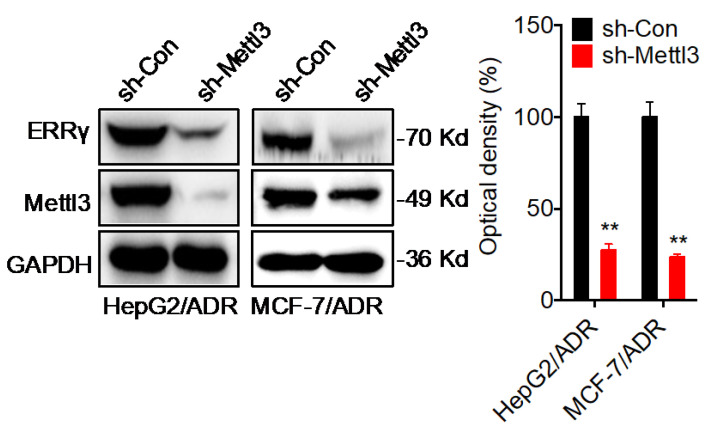
Corrected figure for the original Figure 7 D.

