# Supplementary material for: N6-methyladenosine-induced ERRγ triggers chemoresistance of cancer cells through upregulation of ABCB1 and metabolic reprogramming
Source: Theranostics. 2020 Feb 10;10(8):3382–96. doi: 10.7150/thno.40144 (PMC7069076; doi:10.7150/thno.40144)
Supplement: Supplementary file 1 — Supplementary figures, tables, materials and methods. [file thnov10p3382s1.pdf]

Supplementary data for

N6-methyladenosine-induced ERRγ triggers chemoresistance of cancer cells through upregulation of ABCB1 and metabolic reprogramming

Chen et al

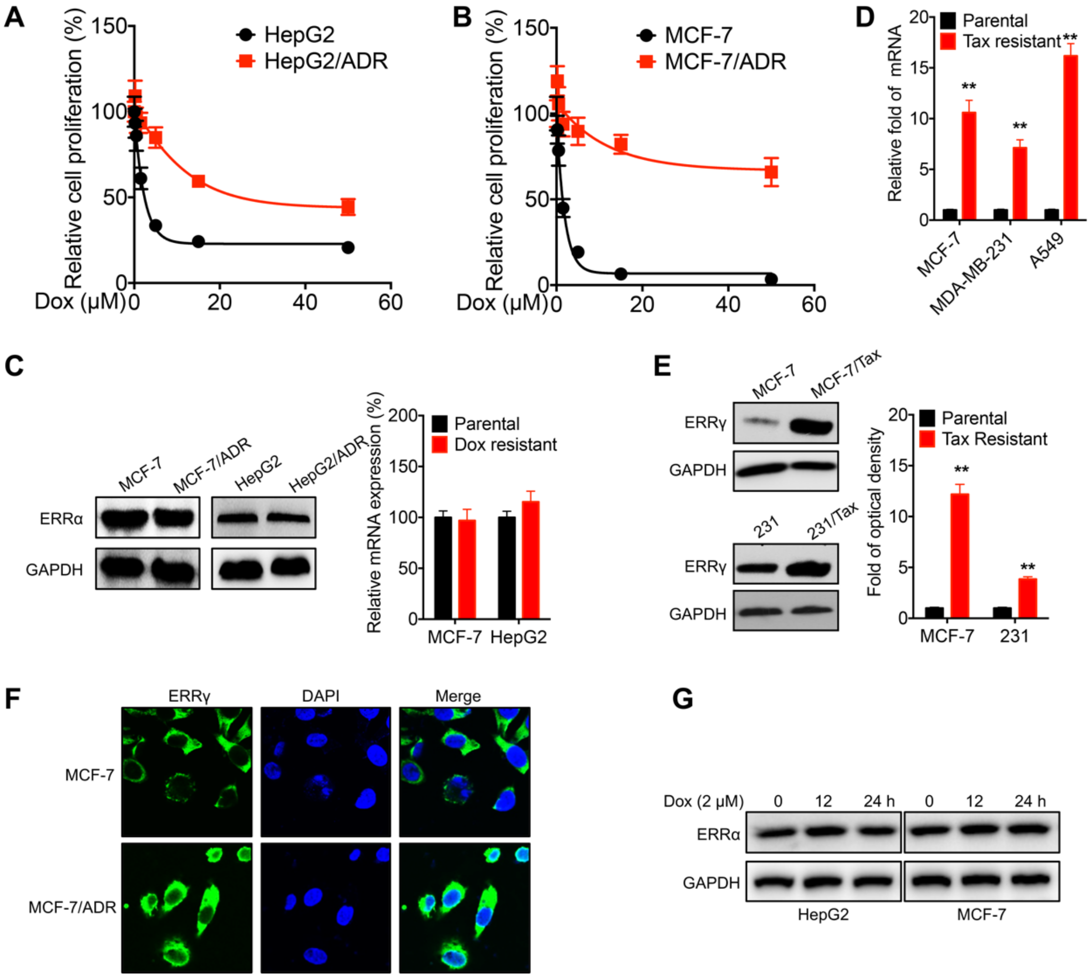

Figure S1 ERRγ is upregulated in chemoresistant cancer cells.

(A&B) Cell proliferation measured in HepG2/ADR (A) or MCF-7/ADR (B) and their corresponding parental cells treated with increasing concentrations of Dox for 48 h;

(C) Protein expression of ERRα in HepG2/ADR, MCF-7/ADR cells, and their corresponding parental cells measured by Western blot analysis (left) and quantitatively analyzed (right);

(D) The mRNA expression of ERRγ was measured in MCF-7/Tax, MDA-MB-231/Tax, A549/Tax and their corresponding parental cells by qRT-PCR;

(E) The protein expression of ERRγ in MCF-7/Tax, MDA-MB-231/Tax, and their corresponding parental cells was checked by Western blot analysis;

(F) The subcellular expression of ERRγ in MCF-7/Dox and MCF-7 cells was checked by confocal imaging;

(G) Cells were treated with Dox (2 μM) for the indicated time, then protein expression of ERRα was checked by Western blot analysis.

Data were presented as means ± SD from three independent experiments. ***p*< 0.01 compared with control.

Related to Figure 1

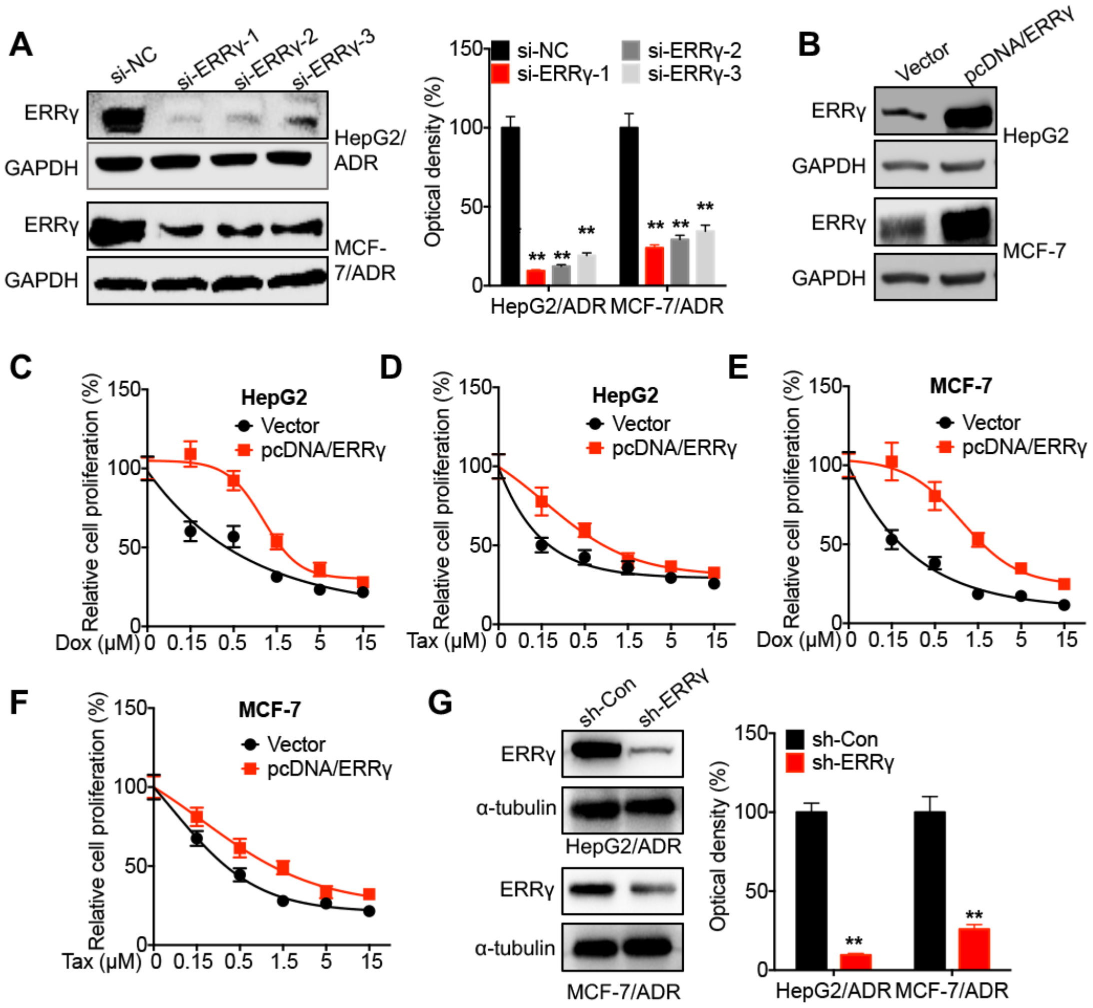

Figure S2 ERRγ regulates chemoresistance of cancer cells.

(A) Cells were transfected with si-NC or si-ERRγ-1/2/3 for 24 h. The expression of ERRγ was checked by Western blot analysis (left) and quantitatively analyzed (right). The si-ERRγ-1/2 were used for next studies due to the relative high efficiency;

(B) Cells were transfected with vector control or pcDNA/ERRγ for 24 h and the expression of ERRγ was checked by Western blot analysis;

(C&D) Cell proliferation rate of HepG2 cells transfected with vector control or pcDNA/ERRγ for 24 h, followed by treatment with increasing concentrations of Dox (C) or Tax (D)

for 48 h;

(E&F) Cell proliferation rate of MCF-7 cells transfected with vector control or pcDNA/ERRγ for 24 h, followed by treatment with increasing concentrations of Dox (E) or Tax (F) for 48 h;

(G) Protein levels of ERRγ in cells transfected with sh-Con or sh-ERRγ was checked by Western blot analysis (left) and quantitatively analyzed (right)..

Data were presented as means ± SD from three independent experiments. **$p < 0.01$.

Related to Figure 2.

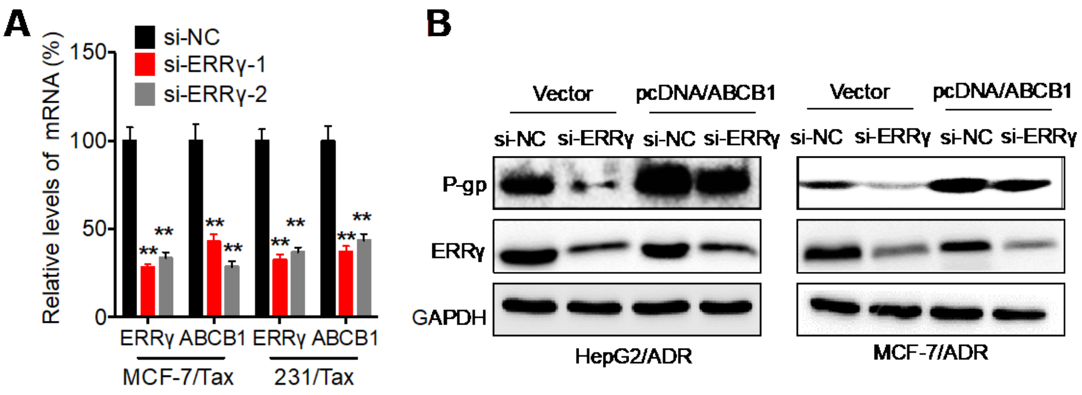

Figure S3 P-gp is involved in ERRγ-regulated chemoresistance of cancer cells.

(A) MCF-7/Tax or MDA-MB-231/Tax cells were transfected with scrambled siRNA or si-ERRγ-1/2 for 24 h and the expression of ERRγ and *ABCB1* was measured by qRT-PCR;

(B) HepG2/ADR or MCF-7/ADR cells were co-transfected with si-NC, si-ERRγ-1, vector control or pcDNA/ABCB1 construct alone or together for 24 h and the expression of ERRγ and P-gp was measured by Western blot analysis.

Data were presented as means ± SD from three independent experiments. **$p < 0.01$. NS, no significant.

Related to Figure 3.

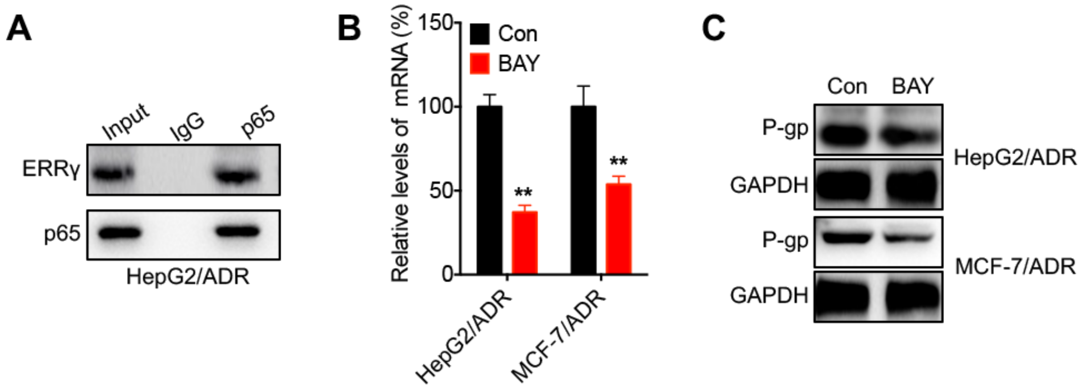

Figure S4 ERRγ interacts with p65 to regulate *ABCB1* transcription.

(A) Interaction between ERRγ and p65 in HepG2/ADR cells was checked by immunoprecipitation using a p65 antibody;

(B&C) HepG2/ADR or MCF-7/ADR cells were treated with or without BAY 11-7082 (10 μM) for 24 h and the mRNA (B) and protein (C) of P-gp were checked by qRT-PCR and Western blot analysis, respectively.

Data were presented as means ± SD from three independent experiments. $^{**}p< 0.01$. NS, no significant.

Related to Figure 4

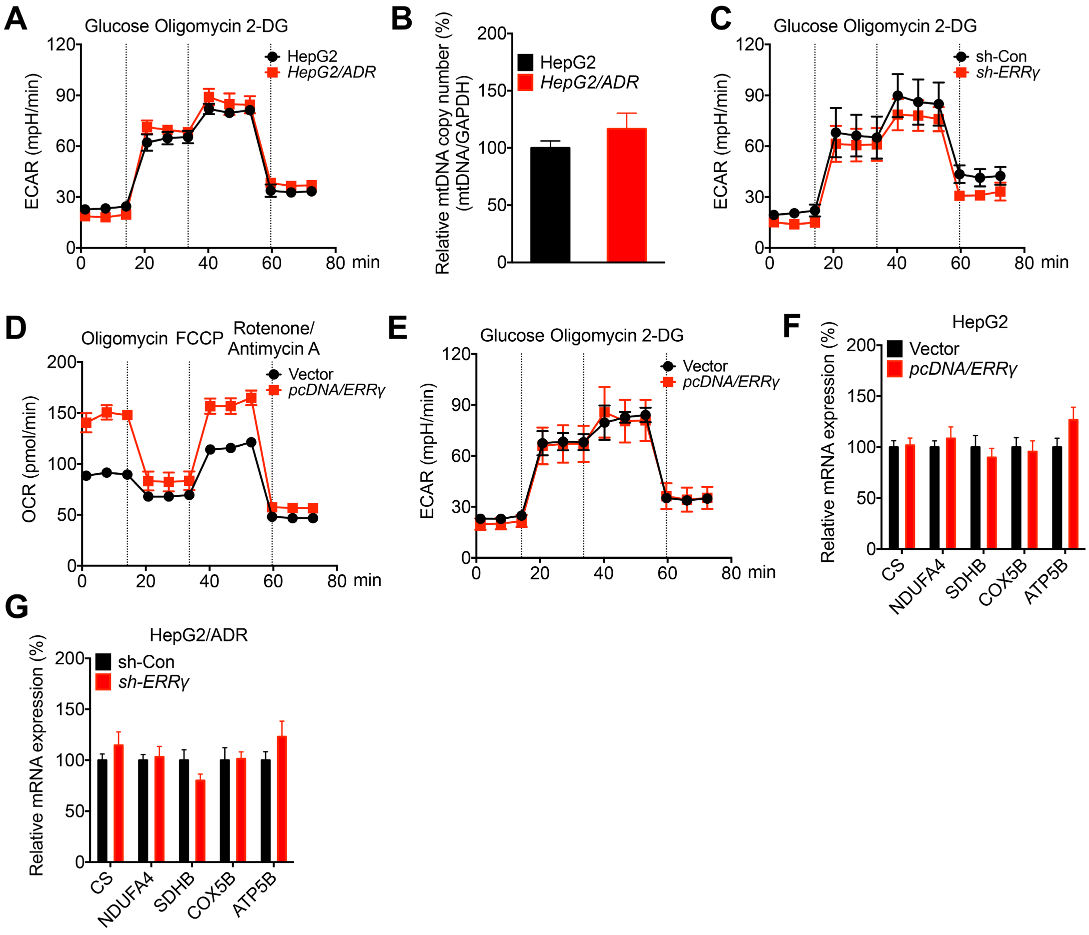

Figure S5 ERRγ dictates the metabolic reprogramming in chemoresistant cancer cells.

(A) Extracellular acidification rate (ECAR) of HepG2/ADR or HepG2 cells was assessed following the addition of 10 mM glucose, 1 μM oligomycin, and 50 mM 2-Deoxy glucose (2-DG) by use of a Seahorse XF24 analyzer;

(B) The relative mitochondrial mass of HepG2/ADR or HepG2 cells;

(C) ECAR of HepG2/ADR transfected with sh-Con or sh-ERRγ was measured by a Seahorse XF24 analyzer;

(D~E) The OCR (D) or ECAR (E) of HepG2 cells transfected with vector control of pcDNA/ ERRγ for 24 h was measured by a Seahorse XF24 analyzer.

(F) HepG2 cells were transfected with pcDNA or pcDNA/ ERRγ for 24 h and the expression of genes related to OxPhos was checked;

(G) The expression of genes related to OxPhos was checked in HepG2/ADR cells transfected with sh-Con or sh-ERRγ.

Data were presented as means ± SD from three independent experiments. ***p*< 0.01. NS, no

significant.

Related to Figure 5.

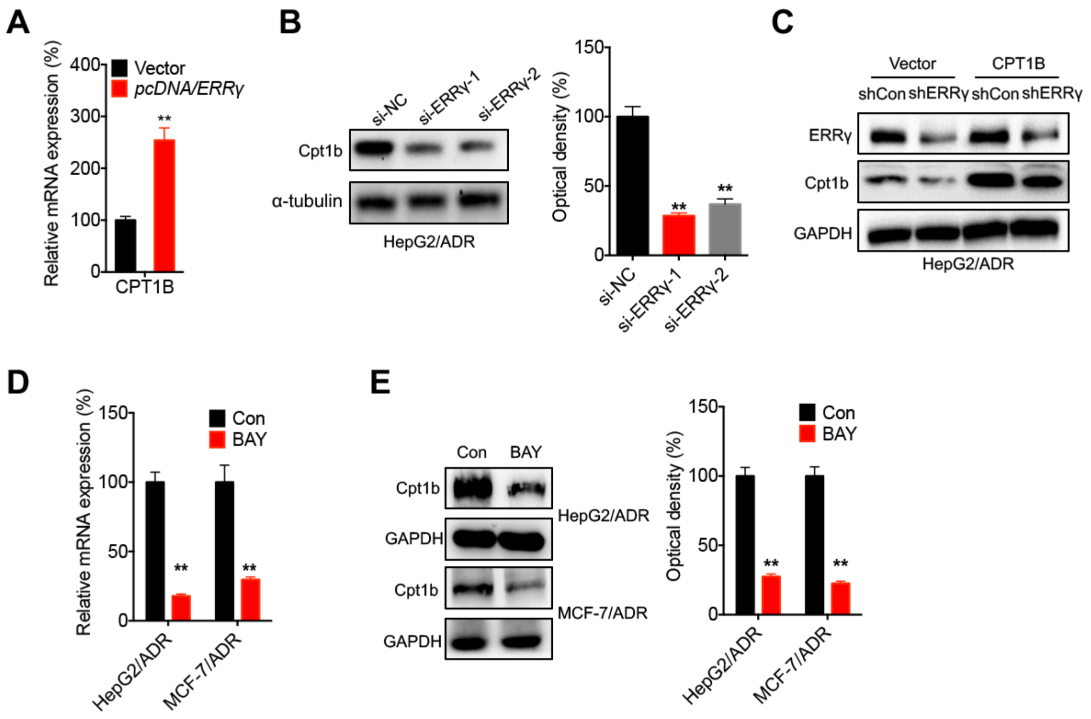

Figure S6 ERRγ regulates the FAO via Cpt1b in chemoresistant cancer cells.

(A) HepG2 cells were transfected with vector or pcDNA/ERRγ for 24 h and the mRNA expression of *CPT1B* was checked by qRT-PCR;

(B) HepG2/ADR cells were transfected with si-NC or si-ERRγ-1/2 for 24 h and the protein expression of Cpt1b was checked by Western blot analysis and quantitatively analyzed;

(C) HepG2/ADR cells transfected with sh-Con or sh-ERRγ were further transfected with vector or CPT1B construct for 24 h and the protein expression was checked by Western blot analysis;

(D&E) HepG2/ADR or MCF-7/ADR cells were treated with or without BAY 11-7082 for 24 h and the mRNA (D) and protein (E) expression of Cpt1b was checked.

Data were presented as means ± SD from three independent experiments. $**p< 0.01$. NS, no significant.

Related to Figure 6.

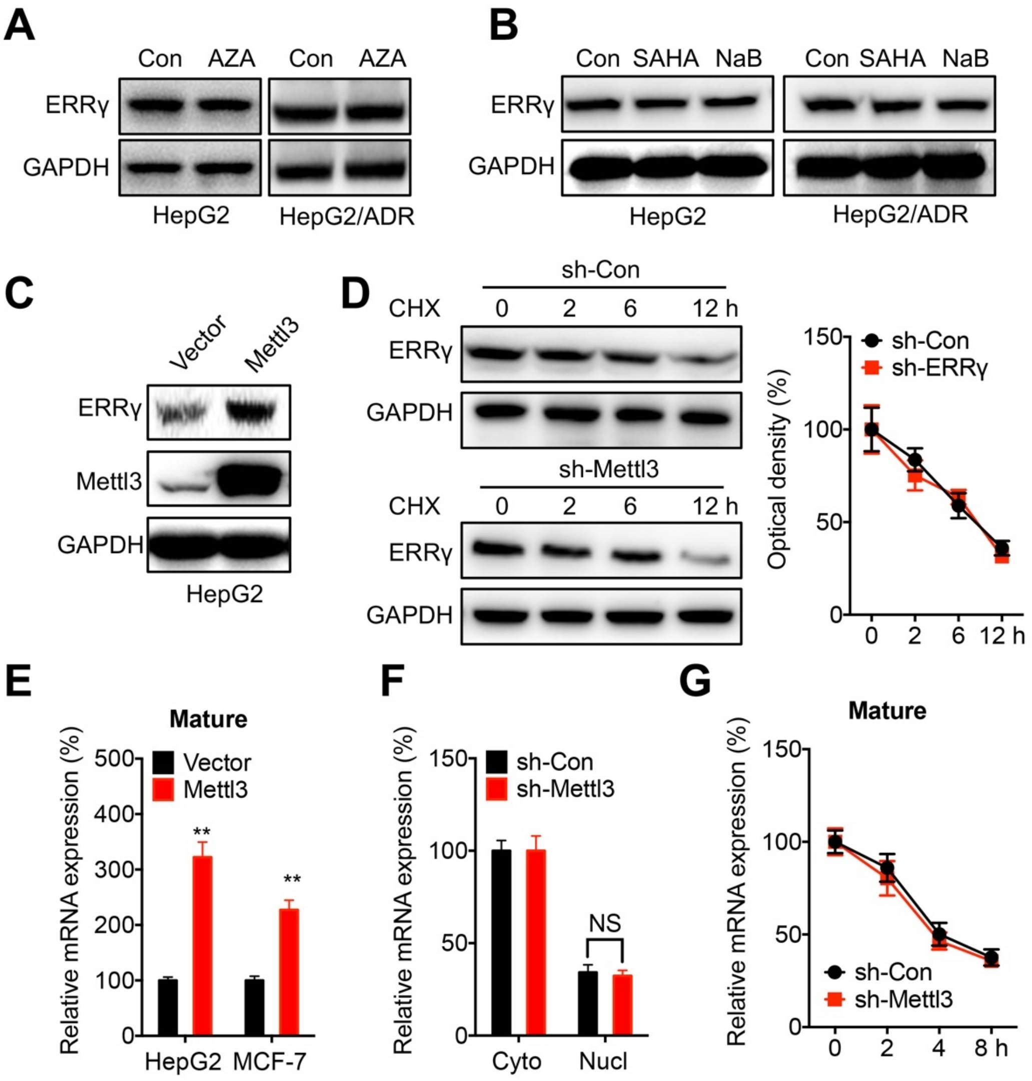

Figure S7 The m^{6}A-facilitated splicing is responsible for the upregulation of ERRγ.

(A) Cells were treated with or without 5 μM 5-aza-dC for 4 days, the expression of ERRγ was tested;

(B) Cells were treated with or without SAHA (2 μM), or NaB (2 mM) for 24 h, the expression of ERRγ was tested;

(C) HepG2 cells were transfected with vector control or Mettl3 constructs for 24 h, the expression of ERRγ was tested;

(D) Cells were treated with CHX for the indicated times, and protein expression of ERRγ was analyzed by western blot analysis (*left*) and quantitatively analyzed (*right*);

(E) Cells were transfected with vector control or Mettl3 constructs for 24 h, the mature mRNA expression of ERRγ was tested;

(F) HepG2/ADR cells were transfected with sh-con or sh-Mettl3 constructs for 24 h, the subcellular localization of mature mRNA of ERRγ was tested;

(G) HepG2/ADR cells transfected with sh-Con or sh-Mettl3 were pre-treated with Act-D for 90 min, then the mature mRNA of ERRγ was checked by qRT-PCR.

Data were presented as means ± SD from three independent experiments. ***p*< 0.01. NS, no significant.

Related to Figure S7.

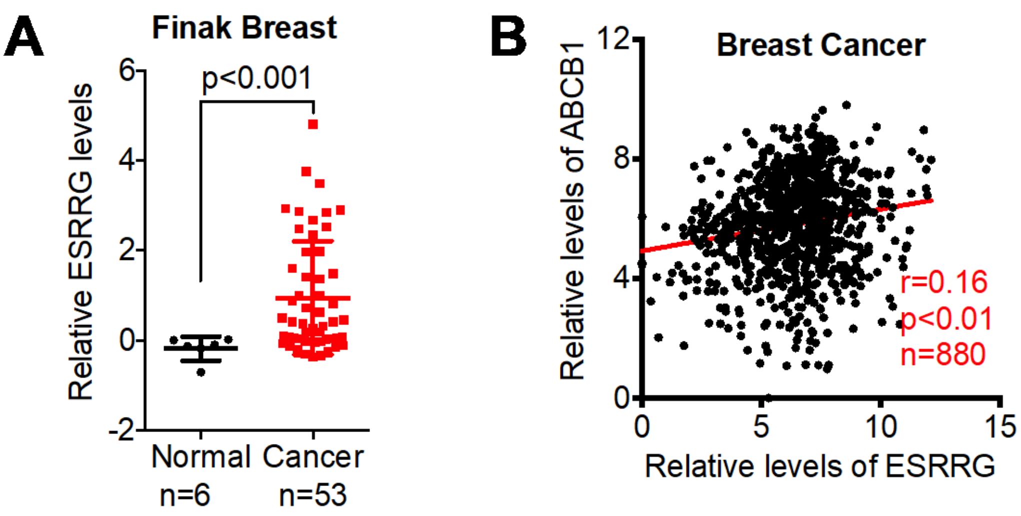

Figure S8 The m^{6}A/ ERRγ axis and *in vivo* cancer progression.

(A) Expression of ESRRG in normal and breast cancer tissues from Oncomine database (Finak breast)

(B) Correlation between ESRRG and ABCB1 in breast cancer patients (n=880) from TCGA database;

Relative to Figure 8.

Supplementary Table 1 The official full name and function of FAO related genes [1]

Catalog	Function	Genes
Acyl-CoA synthetase (long-chain)	Convert free long chain fatty acids into fatty acyl-CoA esters	ACSL4, ACSL5
Carnitine palmitoyltransferase	Promote carnitine dependent transport across the mitochondrial membrane	CPT1A, CPT1B, CPT1C, CPT2
Carnitine O-acetyltransferase	Regulate the acyl-CoA/CoA ratio	CRAT
Acyl-CoA dehydrogenase (C-2 to C-3)	Catalyze the initial step of FAO	ACADS, ACAD9
Acyl-CoA dehydrogenase (C-4 to C-12)	Catalyze the initial step of FAO	ACADM, ACAD10
Acyl-CoA dehydrogenase (long chain)	Catalyze the initial step of FAO	ACADL, ACAD11
Hydroxyacyl-CoA dehydrogenase/3-ketoacyl-CoA thiolase/enoyl-CoA hydratase	Catalyze the last three steps of FAO	HADHA, HADHB
Acetyl-CoA acyltransferase 2	Catalyze the last step of FAO	ACAA2
Enoyl-CoA delta isomerase	Regulate FAO for unsaturated fatty acids	ECI1, ECI2

Table S2 Primers for PCR assay

Gene	Primer sequence
**qRT-PCR**	
ESR1	forward: 5'- GCTTACTGACCAACCTGGCAGA -3'
	reverse: 5'- GGATCTCTAGCCAGGCACATTC -3'
ESR2	forward: 5'- ATGGAGTCTGGTCGTGTGAAGG -3'
	reverse: 5'- TAACACTTCCGAAGTCGGCAGG -3'
ESRRA	forward: 5'- CCACTATGGTGTGGCATCCTGT -3'
	reverse: 5'- GGTGATCTCACACTCGTTGGAG -3'
ESRRB	forward: 5'- GACATTGCCTCTGGCTACCACT -3'
	reverse: 5'- CTCCGTTTGGTGATCTCGCACT -3'
ESRRG	forward: 5'- CGCAGGATAGATGCGGAGAACA -3'
	reverse: 5'- TTCAGCCACCAACAAATGTGAGAC -3'
GPER1	forward: 5'- TTCCGCGAGAAGATGACCATCC -3'
	reverse: 5'- TAGTACCGCTCGTGCAGGTTGA -3'
ABCA1	forward: 5'- CAGGCTACTACCTGACCTTGGT -3'
	reverse: 5'- CTGCTCTGAGAAACACTGTCCTC -3'
ABCB1	forward: 5'- GCTGTCAAGGAAGCCAATGCCT -3'

	reverse: 5’- TGCAATGGCGATCCTCTGCTTC -3’
ABCC1	forward: 5’- CCGTGTACTCCAACGCTGACAT -3’
	reverse: 5’- ATGCTGTGCGTGACCAAGATCC -3’
ABCC2	forward: 5’- GCCAACTTGTGGCTGTGATAGG -3’
	reverse: 5’- ATCCAGGACTGCTGTGGGACAT -3’
ABCC3	forward: 5’- GAGGAGAAAGCAGCCATTGGCA -3’
	reverse: 5’- TCCAATGGCAGCCGCACTTTGA -3’
ABCG2	forward: 5’- GTTCTCAGCAGCTCTTCGGCTT -3’
	reverse: 5’- TCCTCCAGACACACCACGGATA -3’
CS	forward: 5’- CACAGGGTATCAGCCGAACCAA -3’
	reverse: 5’- CCAATACCGCTGCCTTCTCTGT -3’
NDUFA4	forward: 5’- CACTGTATCTCTTGCGTCTGGC -3’
	reverse: 5’- GCTTGCTGTAATCCACATTCACTG -3’
SDHB	forward: 5’- GCAGTCCATAGAAGAGCGTGAG -3’
	reverse: 5’- TGTCTCCGTTCCACCAGTAGCT -3’
COX5B	forward: 5’- GGAGATCATGCTGGCTGCAAAG -3’
	reverse: 5’- GCAGCCTACTATTCTCTTGTTGG -3’
ATP5B	forward: 5’- TCATGCTGAGGCTCCAGAGTTC -3’
	reverse: 5’- ACAGTCTTGCCAACTCCAGCAC -3’
ACAA2	forward: 5’- TCACGAATTAAGGCGTCGAGGTG -3’
	reverse: 5’- TCAGGCTGTGCTCTGAATGATGAC -3’
ACAD10	forward: 5’- CAGCCACAGACGAGCCATATACAC -3’
	reverse: 5’- GCAGCCATTCGATCAGCCTCTC -3’
ACAD11	forward: 5’- AGCACAAGTTCGACAGCAAGTCC -3’
	reverse: 5’- GCAATGGTCAGCGTAGCCTCAC -3’
ACAD9	forward: 5’- GGATCATCTTGGCTGGCACTGAG -3’
	reverse: 5’- TGGCTCCGTGAGGCAGAAGG -3’
ACADL	forward: 5’- CTGTACTCCGCAGCTATTGTCTGG -3’
	reverse: 5’- TCCAGGCTCTGTCATTGCTATTGC -3’
ACADM	forward: 5’- AACATGGGCCAGCGATGTTC -3’
	reverse: 5’- GAAACCAGCTCCGTCACCAA -3’
ACADS	forward: 5’- TGCTGTGAACTACGCTGAGAATCG -3’
	reverse: 5’- TCTGCCAACTTGAACTGGATGACC -3’
ACSL4	forward: 5’- AATGTCCGCATGATGCTGTCTGG -3’
	reverse: 5’- AGGTGCTCCAACTCTGCCAGTAG -3’
ACSL5	forward: 5’- CGGCGTTGATCTGCATCCTGAC -3’
	reverse: 5’- TGGCATCTGAGAAGCAGCAACTTG -3’
CPT1A	forward: 5’- CAGACACCATCCAGCACATGAGAG -3’
	reverse: 5’- TGAGGCTCCGAGGTATTGTCCAG -3’
CPT1B	forward: 5’- ATCCGCATCAAGAATGGCATCCTC -3’
	reverse: 5’- GGAGATGTCCACGTTGCAGAAGG -3’
CPT1C	forward: 5’- CCTTCCAGACCAGATCCACTCCTC -3’

	reverse: 5’- GTGGCAGCGTCGGATGAAGC -3’
CPT2	forward: 5’- GTGATGGTGTGGCAGTGCTCAG -3’
	reverse: 5’- TGGCTGGCTCTGTGGAGTGAC -3’
CRAT	forward: 5’- AGGTGGAGCTGCTGCGGAAG -3’
	reverse: 5’- ATGGCGATGGCGTAGGAGGTG -3’
ECI1	forward: 5’- TCCAACCTGGTGCTGGTCTCC -3’
	reverse: 5’- AGGATGCGGTAGTCACAGGTCAG -3’
ECI2	forward: 5’- CAGGCCACTGAAGGACCTTGTAAC -3’
	reverse: 5’- GGCTCCACCTGACTAGAGGATTCC -3’
HADHA	forward: 5’- CATGGTCAGGTCCTGTGCAAGAAG -3’
	reverse: 5’- CACTTGTTGCTGTCCTCGGTCTAG -3’
HADHB	forward: 5’- CTGACAAGACTCCTGCTCACACTG -3’
	reverse: 5’- CACCACCTGCCACGATCACATC -3’
GAPDH	forward: 5’- ACAACTTTGGTATCGTGGAAGG -3’
	reverse: 5’- GCCATCACGCCACAGTTTC -3’

Materials and Methods

1. Cell lines and cell culture

Human MCF-7, HepG2, MDA-MB-231, and A549 cancer cells were purchased from the American Type Culture Collection (ATCC, Manassas, VA) and maintained in our lab with recommended medium containing 10% fetal bovine serum (FBS) and 1% penicillin/streptomycin (Invitrogen). The HepG2/ADR cells were obtained by treatment with gradually increasing concentration of Adriamycin (ADR) [2]. The MCF-7/ADR was also obtained by treatment with gradually increasing concentration of ADR and kindly provided by Cancer Institute & Hospital Chinese Academy of Medical Sciences (Beijing, China) [3]. The taxol (Tax) resistant MCF-7, MDA-MB-231, and A549 cells were purchased from Shanghai Institute of Cell Resource Center of Life Science (Shanghai, China). DNA fingerprinting was performed using the commercially PowerPlex# 1.2 System kit to confirm the source of chemoresistant cancer cells. Cells were maintained as monolayers in 5% CO_2 at 37 °C. When the cells were 80% confluent, they were sub-cultured to fresh medium. Cells were routinely tested for mycoplasma contamination using MycoAlert Mycoplasma Detection Kit. The cultures were incubated for 24 h before the experimental treatments.

2. qRT-PCR

RNA extraction with Trizol (Invitrogen) and real-time PCR were performed according to the protocols used in our previous study [4]. Quantitative Real-Time PCR (qRT-PCR) was implemented with an iCycler (Bio-Rad, Hercules, USA) using validated primers and SYBR Premix Ex Taq II (Takara, Japan). GAPDH were used as an endogenous control for normalization. Expression levels were calculated using the $2^{-\Delta\Delta Ct}$ method. Three independent experiments were performed. Primer pairs were summarized at Table S2 in the Supplementary Data.

3. Western blot analysis

Western blot analysis was performed according to the protocol used in our previous study [5]. The antibodies used in the present study were: ERRα (Santa Cruz Biotechnology, sc-65715, 1:1000), ERRγ (Santa Cruz Biotechnology, sc-393969, 1:1000), H2A.X (CST, 7631S,

1:1000), P-gp (Bioworld, BS71461, 1:1000), NFκB-p65 (CST, 8242S, 1:1000), c-fos (CST, 2250, 1:1000), c-Jun (CST, 9165, 1:1000), Sp1 (CST, 9389, 1:1000), Cpt1b (Abcam, ab15703, 1:1000), Mettl3 (CST, 96391S, 1:500), GAPDH (BOSTER, BM3876, 1:1000), and α-tubulin (CST, #3873, 1:1000). For measuring the subcellular localization of ERRγ, the nucleus and cytosol were separated by using the NE-PER Nuclear and Cytoplasmic Extraction Kit (Thermo Fisher Scientific, Inc., Pierce, Waltham, MA, USA). GAPDH and H2A.X were used as the loading control for cytosol and nucleus, respectively. GAPDH and α-tubulin were used as the loading control for normalization. The quantitative analysis of Western blot analysis was conducted according to the previous study [6]. Briefly, the intensity of the bands on the blots was measured using Image Lab (Bio-Rad). The intensity values of target proteins were divided by the intensity values of internal control (GAPDH or α-tubulin). The obtained intensity value from control cells was set to 100% with n=3 for each group unless otherwise specified. One of representative Western blot experiments is shown.

4. Immunofluorescence

Immunofluorescent staining was performed as described previously [7]. Briefly, cells growing on the coverslips were washed with PBS, fixed in 4% paraformaldehyde for 15 min and treated with 0.3% triton-x in PBS for 5 min. Cells were blocked with 3% BSA for 1 h at room temperature and incubated with the specific primary antibodies and accordingly dye-conjugated secondary antibody. Finally, cells were counterstained with DAPI (Vector Laboratories, Bulingame, CA). Fluorescent images were acquired using Leica SP8 confocal microscope.

5. Cell proliferation and colony formation

Cell proliferation was tested by CCK-8 kit (Dojindo, Gaithersburg, MD) according to our previous study [7]. Colony formation was detected by CytoSelect 96-well Cell Transformation Assay (Cell Biolabs, USA).

6. Plasmid, siRNA, shRNA and generation of stable cell lines

The small interfering RNA against ERRγ (si-ERRγ) and its negative control (si-NC) were

purchased from RiBo Biotech (Guangzhou RiBo Biotech). Plasmid expressing small hairpin RNA against ERRγ (sh- ERRγ) or Mettl3 (sh-Mettl3) scramble shRNA-encoding plasmid were obtained by GenePharm Co. Ltd. (Shanghai, China). The pcDNA/ERRγ and pcDNA/CPT1B was constructed in our lab by inserting the full length of ERRγ and CTP1B into the pcDNA3.1 vector. Twenty-four hours before transfection, the medium was replaced with fresh medium and transfected using Lipofectamine 2000 reagent (Invitrogen) with vector control, plasmid construct, siRNA negative control (si-NC), or siRNAs according to the manufacturer's instructions. The working concentration of siRNA was 50 nM.

To generate ERRγ knockdown cells, cells were transfected with scramble control or sh- ERRγ lentivirus were selected with 1 μg/ml puromycin for two weeks. The stable cells were cultured in medium supplemented with 1 μg/ml puromycin. Cells were incubated with medium without puromycin for four days before experiments.

7. Rh123 accumulation assay

Rh123 accumulation assay was used to assess the P-gp function according to the previous study [8]. Briefly, cells were treated as indicated conditions and then incubated with Rh123 (10 μM) for 30 min/37°C and analyzed by flow cytometry (FACSCanto, BD Biosciences) at 510 nm excitation/595 nm emission. Data were collected from 10 000 cells per sample.

8. Immunoprecipitation

After lysis and centrifuge, input was done with the 2.5 % of the crude lysate. Equal amounts of ERRγ or p65 were immunoprecipitated with a preclearing process and incubated overnight at 4 ^{o}C with the primary antibody and protein A/G as indicated (SCBT, sc-2003). As control, immunoprecipitation with rabbit IgG (SCBT, sc-11390) was also conducted. After 4 washes with lysis buffer and once with PBS plus inhibitors, pellets were resuspended in 6 x loading buffer, boiled and loaded onto 8 % polyacrylamide gels and transferred to a PVDF membrane (Immobilon-P, Millipore). The expression of interacted proteins was measured by Western blot analysis.

9. Luciferase reporter assay

The promoter activity of ABCB1 or CPT1B was measured according to our previously described protocol [9]. Briefly, the wild type or mutant promoter of ABCB1 or CPT1B were cloned into the pGL3-Basic plasmid and co-transfected with pRL-TK. Transcriptional activity was determined by a luminometer, using a dual luciferase assay kit. Results were displayed as the ratios between the activity of reporter plasmid and pRL-TK.

10. ChIP

The ChIP assay was performed according to our previous study [10]. Briefly, cross-linked and isolated nuclei were sonicated using a Diagenode Bioruptor to an average size of 500 bp in length of treated cells were incubated with 5 μg of anti-ERRγ antibody, rabbit anti-IgG antibody, or no antibody for 16 h at 4°C, followed by incubation with 80 μg of salmon sperm DNA/protein A-agarose for 2 h at 4°C. After washed three times, samples were then treated with RNase A (Roche Diagnostics) for 30 minutes at 37°C and with proteinase K (Roche Diagnostics) for 2 h at 42°C. Isolated DNA fragments were purified with QIAquick spin kit (Qiagen), and quantitative PCRs were performed using 2 μl of DNA in triplicate. The primers were as follow: ABCB1 ERRE1, forward 5'TCA TTT GAA GGT CTT CCC AGT3', reverse 5'TGG CTT AGG GAT TGG GGT AT3'; ABCB1 ERRE2 , forward 5'CCC AAT CCC TAA GCC ATG TA3', reverse 5'GGA GGA AGG GTG GGA GTA GA3'; CPT1B, forward 5' GGA GGA ACA ACG AGC AGA AG3', reverse 5' CCC AGA GCA CTG AAG AGT CC3'.

11. Metabolic assay

The glucose and lactate concentration in cultured media were measured using commercial kits (BioVision) following the manufacturer's instructions and previous study [11]. ATP was quantified using CellTiter-Glo® luminescence assay (Promega) according to the manufacturer's instructions and previous study [12]. All samples were tested in triplicate. PDH activity was measured using the MitoProfile Dipstick Assay Kit (MitoSciences) as previously described [13].

12. ECAR and OCR

Extracellular acidification rate (ECAR) and oxygen consumption rate (OCR). ECAR and

OCR were analyzed on a XF96 Extracellular Flux Analyzer (Seahorse Bioscience) as previously described [14]. Cells were plated in non-buffered DMEM media with 10mM glucose. Measurements were obtained under basal conditions and after the addition of 2 μM oligomycin and 100mM 2-DG.

13. Mitochondrial DNA assay

Mitochondrial DNA (mtDNA) copy number was determined as previously described [15]. Briefly, cells relative content of mtDNA (mtDNA primer set) was checked by qRT-PCR and normalized to that of nuclear DNA (GAPDH primer set). The primers were as follow: mtDNA, forward 5' ACGCCATAAAACTCTTCACCAAAG', reverse 5' GGGTTCATAGTAGAAGAGCGATGG3'; GAPDH, forward 5' ACAACTTTGGTATCGTGGAAGG', reverse 5' GCCATCACGCCACAGTTTC 3';

14. Fatty acid uptake assay

The fatty acid uptake assay was conducted by use of a QBT™ Fatty acid uptake assay kit (Molecular Devices) according to the previous study [16]. Briefly, after extracellular FAs were washed away, cells were followed by the addition of QBT FA-loading buffer to each well. The fluorescence intensity was measured on the PowerScan HT microplate reader. Instrument settings were bottom read, excitation 488/emission 515, with a filter cutoff at 495 nm.

15. Fatty acid oxidation assay

The mitochondria of cells were isolated by using the Cell Mitochondrial Isolation Kit (Beyontime, Jiangsu, China). Then the fatty acid β-oxidation rate was measured by use of the Fatty Acid β-Oxidation Kit from Genmed Scientifics Int USA according to the previous study [17].

16. LC-MS/MS assay for m^{6}A

LC-MS/MS assay for m6A quantification was conducted according to our recent study [18]. Briefly, mRNA was purified from total RNA using oligo dT magnetic beads and incubated

with nuclease P1 (0.5 U, Sigma) and alkaline phosphatase (1 μL, 1 U/μL; Sigma) to digestion. All samples (10 μl for each injection) were separated by a C18 column (Agilent) using reverse-phase ultra-performance liquid chromatography and analyzed by an Agilent 6410 QQQ triple-quadrupole LC mass spectrometer using positive electrospray ionization mode. All nucleosides were quantified by use of retention time and ion mass transitions of 268.0 to 136.0 (A) and 282.1 to 150.0 (m^6A). Quantification was calculated using standard curves from standards running in the same batch. Ratio of m^6A to A was calculated based on calibration curves.

17. RNA stability

To measure RNA stability, actinomycin D (Act-D, Catalog #A9415, Sigma, U.S.A) at 5 μg/ml was added to cells. After incubation at the indicated times, cells were collected and RNA was isolated for Real time PCR. Half-life (t1/2) of precursor and mature RNA were calculated using ln2/ slope.

18. Protein stability

To measure protein stability, cells were treated with cycloheximide (CHX, final concentration 100 μg/ml) during indicated times. The expression of ERRγ was measured through western blot analysis.

19. Subcellular fraction of RNA

The nucleus and cytoplasm were separated by NE-PER nuclear and cytoplasmic extraction reagents (Pierce, Rockford, IL, USA). The fractions were pooled to isolate total RNA by TRIzol reagent for RT-PCR.

20. Protein stability

To measure protein stability, cells were treated with cycloheximide (CHX, final concentration 100 μg/ml) during indicated times. The expression of ERRγ was measured through western blot analysis.

21. Experimental animals and xenograft models

BALB/c nude mice (four weeks old) were purchased from Sun Yat-sen University (Guangzhou, China) Animal Center and raised under pathogen-free conditions. All animal experiments complied with Zhongshan School of Medicine Policy on Care and Use of Laboratory Animals. Both sh-control and *sh*- ERRγ HepG2/ADR cells (5×10^6 per mouse, n=5 for each group) were diluted in 200μL PBS + 200 μL Matrigel (BD Biosciences) and subcutaneously injected into immunodeficient mice. After one week, mice were injected i.v. (bolus) through a tail vein at a 3-day interval with saline solution or Dox in saline solution. The injection volume (approximately 0.1 ml/10g body weight) of micelle solution was adjusted to give 10 mg Dox/kg body weight. Tumor volume and mice weight were monitored with relapsed time. The tumor volume was calculated using the formula V=1/2× larger diameter × (smaller diameter)^2.

22. Immunohistochemistry (IHC)

Immunohistochemistry was performed to measure expression of target protein according to our previous study [19]. The protein expression was assessed semi-quantitatively by two of the authors. The intensity was scored on a scale of 0 - 3 as negative (0), weak (1), medium (2) or strong (3). The extent of the staining, defined as the percentage of positive stained areas of tumor cells per the whole tumor area, was scored on a scale of 0 (0%), 1 (1-25%), 2 (26-50%), 3 (51-75%) and 4 (76-100%). An overall protein expression score (overall score range, 0 to 12) was calculated by multiplying the intensity and positivity scores.

23. Database (DB) search

Data about the expression of ESRRG/METTL3 in liver cancer and normal tissues were further obtained from the Oncomine database (www.oncomine.org) as follows: Guichard liver, TCGA liver, Roessler liver, and Finak breast. The expression profiles of ESRRG among the T stages of liver cancer in patients and its association with ABCB1 were downloaded from LinkedOmics (http://www.linkedomics.org), which is a publicly available portal that includes multi-omics data from all 32 cancer types from TCGA. The LinkedOmics website allowed a flexible exploration of associations between a molecular or clinical attribute of interest and all

other attributes, providing the opportunity to analyse and visualize associations between billions of attribute pairs for each cancer cohort [20].

24. Statistical analyses

Data was reported as mean ± SD from three independent experiments. Data was analyzed by two-tailed unpaired Student's t-test between two groups and by One-Way ANOVA followed by Bonferroni test for multiple comparison. *p<0.05, **p< 0.01; NS, no significant.

References

1. Tan Z, Xiao L, Tang M, Bai F, Li J, Li L, et al. Targeting CPT1A-mediated fatty acid oxidation sensitizes nasopharyngeal carcinoma to radiation therapy. Theranostics. 2018; 8: 2329-47.
2. Li J, Liu J, Guo N, Zhang X. Reversal of multidrug resistance in breast cancer MCF-7/ADR cells by h-R3-siMDR1-PAMAM complexes. Int J Pharm. 2016; 511: 436-45.
3. Yuan WQ, Zhang RR, Wang J, Ma Y, Li WX, Jiang RW, et al. Asclepiasterol, a novel C-21 steroidal glycoside derived from Asclepias curassavica, reverses tumor multidrug resistance by down-regulating P-glycoprotein expression. Oncotarget. 2016; 7: 31466-83.
4. Chen Z, Qi M, Shen B, Luo G, Wu Y, Li J, et al. Transfer RNA demethylase ALKBH3 promotes cancer progression via induction of tRNA-derived small RNAs. Nucleic Acids Res. 2019; 47: 2533-45.
5. Zhou Y, Lu L, Jiang G, Chen Z, Li J, An P, et al. Targeting CDK7 increases the stability of Snail to promote the dissemination of colorectal cancer. Cell Death Differ. 2019; 26: 1442-52.
6. Tojkander S, Ciuba K, Lappalainen P. CaMKK2 Regulates Mechanosensitive Assembly of Contractile Actin Stress Fibers. Cell Rep. 2018; 24: 11-9.
7. Wei W, Chen ZJ, Zhang KS, Yang XL, Wu YM, Chen XH, et al. The activation of G protein-coupled receptor 30 (GPR30) inhibits proliferation of estrogen receptor-negative breast cancer cells in vitro and in vivo. Cell Death Dis. 2014; 5: e1428.
8. Seebacher NA, Richardson DR, Jansson PJ. Glucose modulation induces reactive oxygen species and increases P-glycoprotein-mediated multidrug resistance to chemotherapeutics. Brit J Pharmacol. 2015; 172: 2557-72.
9. Jiang GM, Wang HS, Zhang F, Zhang KS, Liu ZC, Fang R, et al. Histone deacetylase inhibitor induction of epithelial-mesenchymal transitions via up-regulation of Snail facilitates cancer progression. Biochim Biophys Acta. 2013; 1833: 663-71.
10. Wu YM, Chen ZJ, Liu H, Wei WD, Lu LL, Yang XL, et al. Inhibition of ERRalpha suppresses epithelial mesenchymal transition of triple negative breast cancer cells by directly targeting fibronectin. Oncotarget. 2015; 6: 25588-601.
11. Wang LY, Hung CL, Chen YR, Yang JC, Wang J, Campbell M, et al. KDM4A Coactivates E2F1 to Regulate the PDK-Dependent Metabolic Switch between Mitochondrial Oxidation and Glycolysis. Cell Rep. 2016; 16: 3016-27.
12. Lucantoni F, Dussmann H, Llorente-Folch I, Prehn JHM. BCL2 and BCL(X)L selective inhibitors decrease mitochondrial ATP production in breast cancer cells and are synthetically

lethal when combined with 2-deoxy-D-glucose. Oncotarget. 2018; 9: 26046-63.
13. Al Batran R, Gopal K, Aburasayn H, Eshreif A, Almutairi M, Greenwell AA, et al. The antianginal ranolazine mitigates obesity-induced nonalcoholic fatty liver disease and increases hepatic pyruvate dehydrogenase activity. JCI Insight. 2019; 4.
14. Li L, Li L, Li W, Chen T, Bin Z, Zhao L, et al. TAp73-induced phosphofructokinase-1 transcription promotes the Warburg effect and enhances cell proliferation. Nat Commun. 2018; 9: 4683.
15. Rooney JP, Ryde IT, Sanders LH, Howlett EH, Colton MD, Germ KE, et al. PCR based determination of mitochondrial DNA copy number in multiple species. Methods Mol Biol. 2015; 1241: 23-38.
16. Ladanyi A, Mukherjee A, Kenny HA, Johnson A, Mitra AK, Sundaresan S, et al. Adipocyte-induced CD36 expression drives ovarian cancer progression and metastasis. Oncogene. 2018; 37: 2285-301.
17. Tan Y, Lin K, Zhao Y, Wu Q, Chen D, Wang J, et al. Adipocytes fuel gastric cancer omental metastasis via PITPNC1-mediated fatty acid metabolic reprogramming. Theranostics. 2018; 8: 5452-68.
18. Lin X, Chai G, WU Y, Li J, Chen F, Liu J, et al. RNA m6A methylation regulates the epithelial mesenchymal transition of cancer cells and translation of Snail. Nat Commun. 2019: doi.org/10.1038/s41467-019-09865-9.
19. Chen ZJ, Wei W, Jiang GM, Liu H, Wei WD, Yang X, et al. Activation of GPER suppresses epithelial mesenchymal transition of triple negative breast cancer cells via NF-kappaB signals. Mol Oncol. 2016; 10: 775-88.
20. Vasaikar SV, Straub P, Wang J, Zhang B. LinkedOmics: analyzing multi-omics data within and across 32 cancer types. Nucleic Acids Res. 2018; 46: D956-D63.
